# Electrostatic Map Of Proteasome α-Rings Encodes The Design of Allosteric Porphyrin-Based Inhibitors Able To Affect 20S Conformation By Cooperative Binding

**DOI:** 10.1038/s41598-017-17008-7

**Published:** 2017-12-06

**Authors:** Antonio Di Dato, Alessandra Cunsolo, Marco Persico, Anna Maria Santoro, Alessandro D’Urso, Danilo Milardi, Roberto Purrello, Manuela Stefanelli, Roberto Paolesse, Grazia R. Tundo, Diego Sbardella, Caterina Fattorusso, Massimo Coletta

**Affiliations:** 1 Dipartimento di Farmacia Università di Napoli “Federico II”, Via D. Montesano, 49 I, 80131 Napoli, Italy; 2 Dipartimento di Scienze Chimiche, Università degli Studi di Catania, Viale A. Doria 6, 95125 Catania, Italy; 3 Istituto di Biostrutture e Bioimmagini–CNR sede secondaria di Catania, Via P. Gaifami, 9- 95126 Catania, Italy; 4 Dipartimento di Scienze e Tecnologie Chimiche, Università di Roma Tor Vergata-Via della Ricerca Scientifica, 00133 Roma, Italy; 5 Dipartimento di Scienze Cliniche e Medicina Traslazionale, Università di Roma Tor Vergata, Via Montpellier 1, 00133 Roma, Italy

## Abstract

The importance of allosteric proteasome inhibition in the treatment of cancer is becoming increasingly evident. Motivated by this urgent therapeutic need, we have recently identified cationic porphyrins as a highly versatile class of molecules able to regulate proteasome activity by interfering with gating mechanisms. In the present study, the mapping of electrostatic contacts bridging the regulatory particles with the α-rings of the human 20S proteasome led us to the identification of (meso-tetrakis(4-N-methylphenyl pyridyl)-porphyrin (pTMPyPP4) as a novel non-competitive inhibitor of human 20S proteasome. pTMPyPP4 inhibition mechanism implies a positive cooperative binding to proteasome, which disappears when a permanently open proteasome mutant (α-3ΔN) is used, supporting the hypothesis that the events associated with allosteric proteasome inhibition by pTMPyPP4 interfere with 20S gating and affect its “open-closed” equilibrium. Therefore, we propose that the spatial distribution of the negatively charged residues responsible for the interaction with regulatory particles at the α-ring surface of human 20S may be exploited as a blueprint for the design of allosteric proteasome regulators.

## Introduction

The human proteasome is a supramolecular protein assembly with a key role in the intracellular degradation of proteins. This multiprotein complex consists of a 20S proteolytic core capped with different types of regulatory particles (RPs) which are deputed to unfold protein-substrates and kindle proteolytic activity^1–3^. The 20S core particle (CP) is made up by four packed rings: two α subunit rings and two central β subunit rings, each being constituted by seven distinct proteins, with the N-terminal tails of the α subunits composing the substrate access gate. In the β rings there are three different proteolytically active subunits exhibiting caspase-like (PGPH-L; β1), trypsin-like (T-L; β2), and chymotrypsin-like (ChT-L; β5) activity. The α-rings play a structural function, stabilizing the β rings, being also responsible for the interaction with RPs^4^. By anchoring to the α ring surface, RPs not only regulate the access of proteasome substrates to the catalytic chamber regulating the open-closed equilibrium of the gate^5,6^, but also influence the proteasome catalytic performance by allosteric signaling^7,8^. However, it has been reported that 20S CP is able to perform proteolytic activity even without the assistance of RPs^9,10^. In particular, by applying NMR, as well as imaging AFM techniques, in combination with biochemical and mutational studies, it was demonstrated^11–13^ that the 20S CP spontaneously interconverts between the open and the closed state via multiple conformations and that changes in their populations lead to differences in substrate proteolysis patterns. The authors proved that the relative populations of these conformers are shifted not only by RP binding or mutation of residues that contact RP, but also by substrate/inhibitors binding or mutations of the catalytic β subunits which induce conformational changes to the RP binding site, thus envisaging the existence of a feedback loop between catalysis and gate opening.

The proteasome is hyperactive in malignant tumors and cancer cells are more susceptible than healthy cells to the blocking of its proteolytic activity^14–16^. Therefore, the human 20S has emerged over the past decade^17^ as an attractive target in cancer therapy with the catalytic site inhibitor Bortezomib approved as a revolutionary therapy for multiple myeloma^18^. Despite the initial enthusiasm for the efficacy of these treatments, clinicians had to face soon the problem of relapse, as often cancer patients developed drug resistance^19,20^. Furthermore, Bortezomib is active against only a narrow range of blood malignancies, and possesses severe side effects^21,22^. A possible improvement of proteasome-targeting drugs action should be based on alternative, and more sophisticated, inhibition mechanisms, e.g. targeting non-catalytic sites of the 20S particle and modulating its activity by allosteric signaling^12,13,23^. This approach has already proven to be successful, as shown by recent reports on some Arg/Lys-rich peptides, such as the octa peptide Arg(8)^24^, the natural antibacterial cathelicidin peptide PR39^25^, and the peptide Tat, a fragment of the basic domain of HIV-Tat1 protein, which inhibits proteasome through binding to the α-face^26,27^.

**Figure 1:**
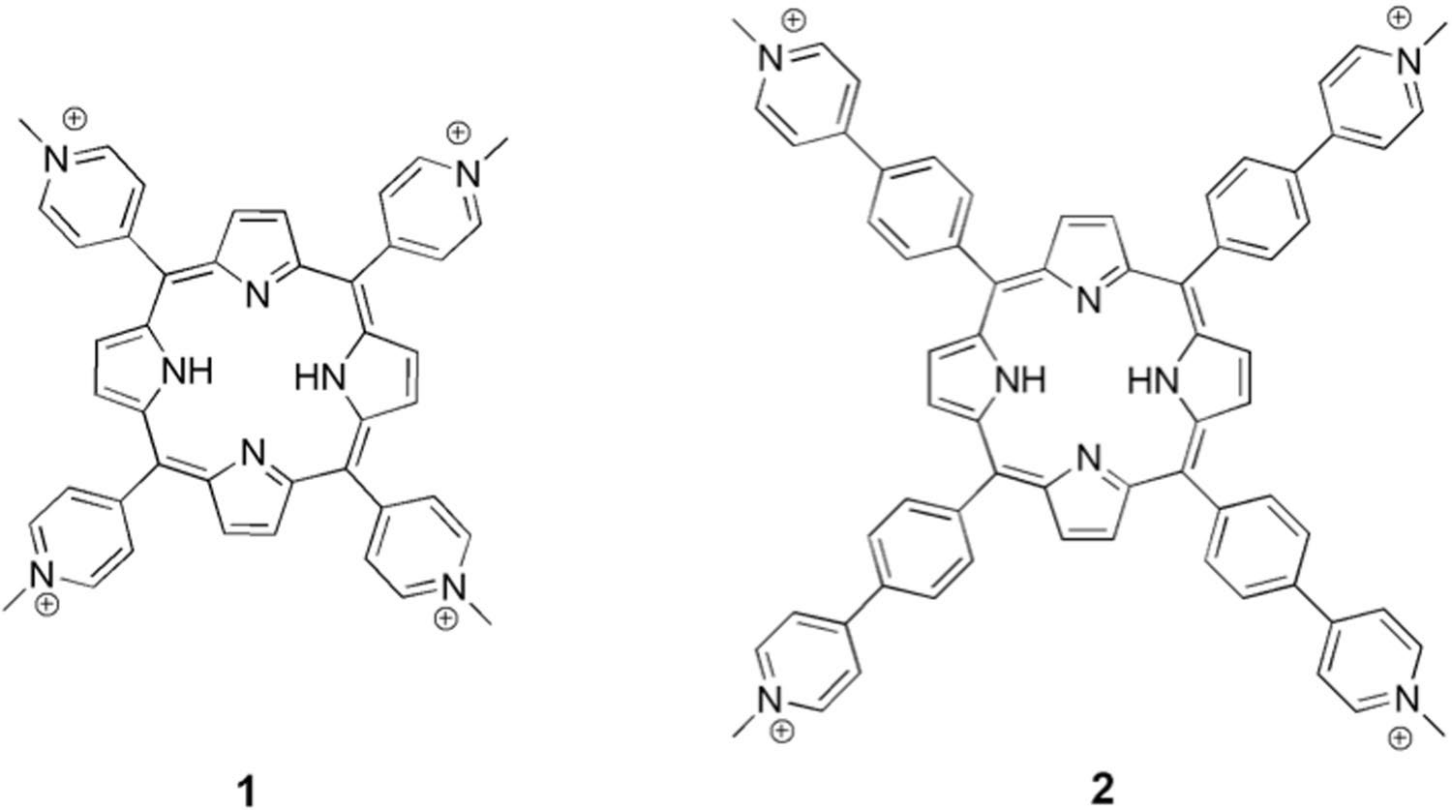
Chemical structures of cationic porphyrins H_2_T4 (**1**) and pTMPyPP4 (**2**).

In line with these findings, we recently identified the cationic porphyrin H_2_T4 (**1**, Fig. 1) as a new lead structure for the development of a novel class of multifunctional inhibitors^28^. In fact, as suggested by docking and confirmed by NMR measurements, **1** acts as a competitive and reversible inhibitor of the human 20S proteasome^29^ thanks to the interactions of the positively charged N-methyl-pyridyl moieties with the negative residues on the α-ring in proximity to the gate channel.

The key role played by electrostatics in driving porphyrin/proteasome interactions suggested that these highly versatile inhibitors may be fine-tuned by conjugating the parent frame with different charged moieties endowing special properties to the molecule. However, we are still far from a complete understanding of the structural features required to generate a fully allosteric porphyrin-based proteasome inhibitor.

To fill this gap, here we mapped the negatively charged residues of the human 20S involved in ionic interactions with the positively charged residues of three canonical human 20S proteasome RPs (PA28α, PA200, and 19S RPs), with the aim of finding putative binding sites for cationic porphyrin-based allosteric inhibitors. Indeed, ligand binding to such negative residues may affect the allosteric mechanism connecting the opening/closing of the gate with the activation of proteasome proteolytic activity^30^. Accordingly, we designed and synthesized a novel porphyrin having in the meso positions four phenylpyridin moieties (meso-tetrakis(4-N-methylphenyl pyridyl)-porphyrin; pTMPyPP4 (**2**) (see Figure 1), resulting in a frame with the positive charges distanced of about 17 Å rather than 11 Å of the parental molecule **1**. The interaction of **2** with the human 20S proteasome was investigated by steady-state and stopped flow kinetic experiments. Our results show an allosteric inhibition of the 20S catalytic activities by **2**, highlighting, in addition, a cooperative binding of at least 3 inhibitor molecules and disclosing a possible connection between the inhibition mechanism of **2** and the allosteric mechanism of activating RPs. Noteworthy, experimental results indicate drastic changes of the inhibition mechanism of **2** in the presence of 20S α-3ΔN yeast mutant proteasome, locked in a permanently open conformation.

## Results and Discussion

### Mapping the negatively charged residues involved in ionic interactions with RPs on human 20S proteasome

First, we performed a bioinformatics study to identify the negatively charged residues of the human 20S CP involved in ionic interactions with positively charged residues of the RPs (see SI for details).

**Figure 2:**
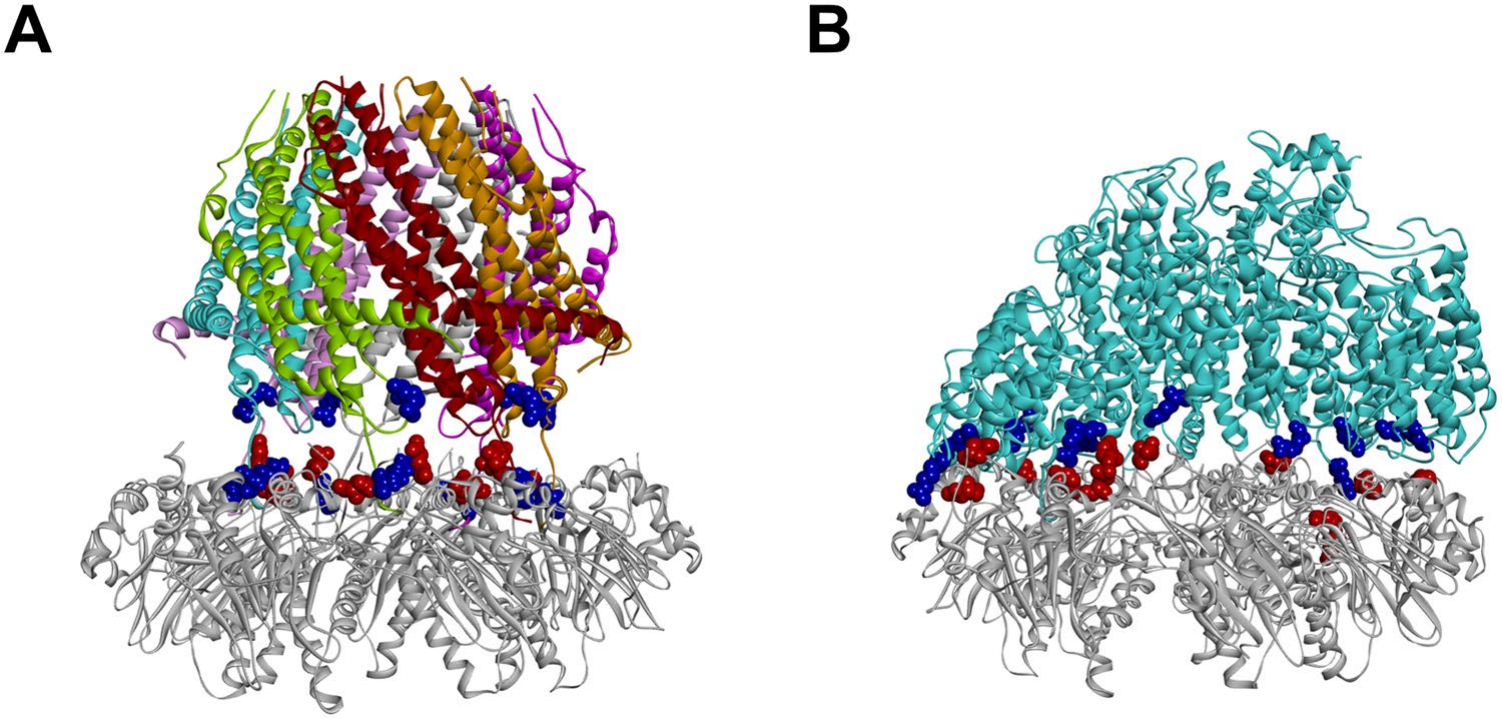
Homology models of human 20S (α-ring) in complex with PA28 (**A**) and PA200 (**B**). The residues involved in ionic interactions are evidenced (CPK render) and colored in red (negative, 20S) and blue (positive, PA28 and PA200). Proteins are displayed as solid ribbons; PA28 (**A**) is colored by subunit type.

In this study, we considered the three canonical human 20S proteasome RPs: PA28α (REGα/11 S), PA200, and 19S. PA28 is an ATP-independent activating complex of human 20S consisting in seven protein subunits with three identified isoforms (PA28α-γ)^31^. In order to map the interactions of the human 20S with PA28, the X-ray complex of *Saccharomyces cerevisiae* 20S with *Trypanosoma Brucei* PA26 (homolog of human PA28^32^) was used as reference structure. The residues of the complex involved in ionic interactions between the CP (negative) and the RPs (positive) were identified. Finally, the information was transferred to the corresponding human homologs using the X-ray structures of PA28α and human 20S (closed and open states) (Fig. 2A; Table S1).

PA200 is also an ATP-independent activator, stimulating PGPH-L proteasomal hydrolysis almost three times more than ChT-L and T-L activity^33^. The ionic interactions occurring between PA200 and human 20S (open and closed states) were identified using the X-ray complex of *Saccharomyces cerevisiae* 20S with the RP Blm10^8^ (yeast homolog of human PA200) as reference structure (Fig. 2B and Table S2).

**Figure 3:**
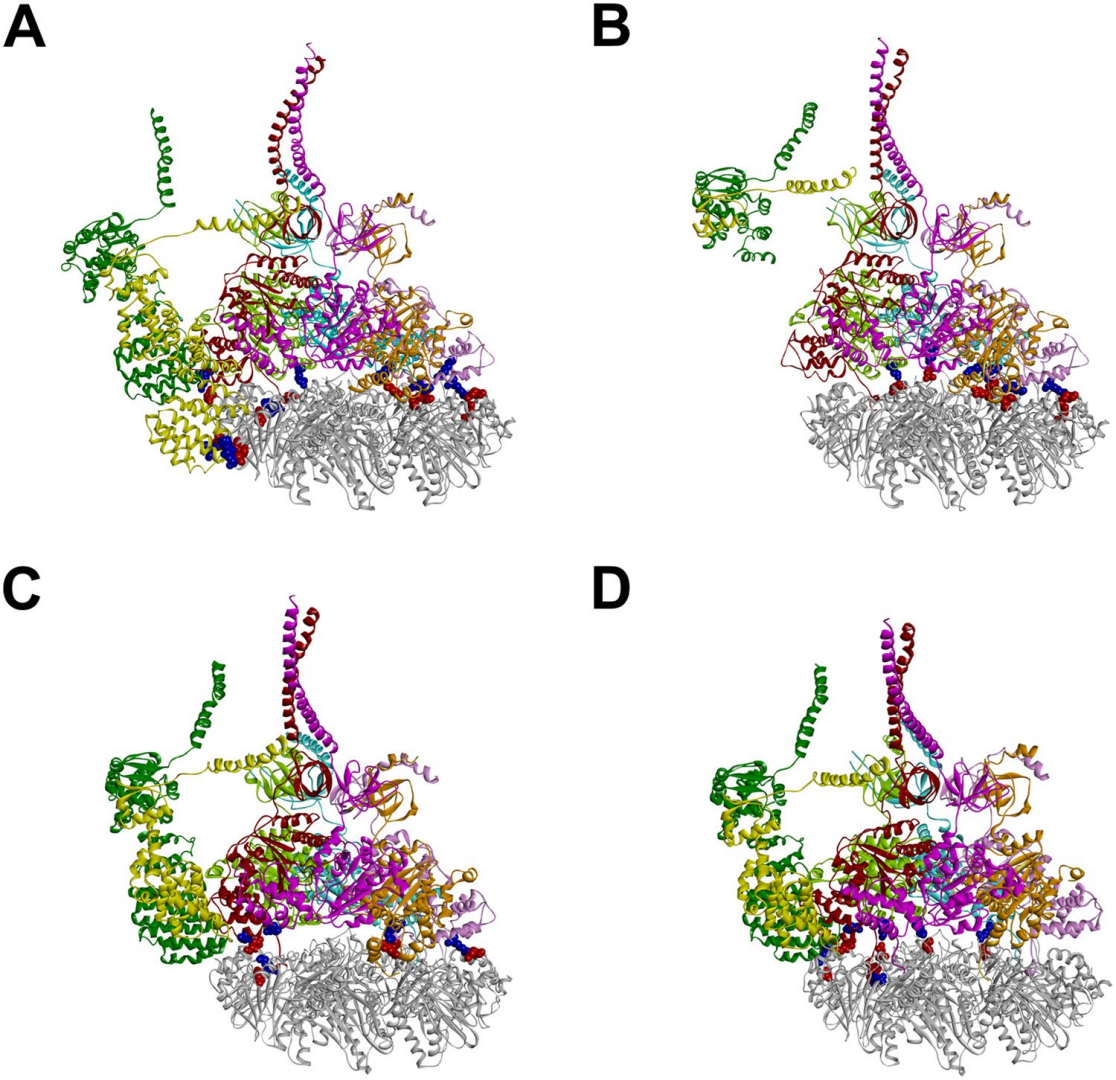
Ionic interactions occurring between human 20S and 19S according to Cryo-EM data^35^. Side view of: S_A_, S_B_, S_C_, and S_D_ conformational/functional states. The residues involved in ionic interactions between 20S (negative: red) and 19S (positive: blue) are displayed as CPK. Proteins are displayed as solid ribbons and colored as follows: 20S α-ring (gray), 19S Rpt1 (pink), Rpt2 (orange), Rpt3 (brown), Rpt4 (light green), Rpt5 (cyan), Rpt6 (magenta), Rpn5 (green), and Rpn6 (yellow).

Finally, the 19S is an ATP-dependent RP complex which induces a ∼3-fold stimulation of the human 20S proteasome three catalytic activities^34^. It is composed by several ATPase and non-ATPase subunits which are organized in a cylinder shaped assembly. Recently, using Cryo-EM techniques^35^, solved the structures of four different 20S-19S complexes (named S_A-D_), from the ground state (S_A_) to the opening of the 20S gate channel (S_D_). The four complex structures were used in the present analysis to investigate the ionic interactions occurring at 20S-19S interface (Fig. 3; see Supplementary Fig. S3; Table S3).

**Figure 4:**
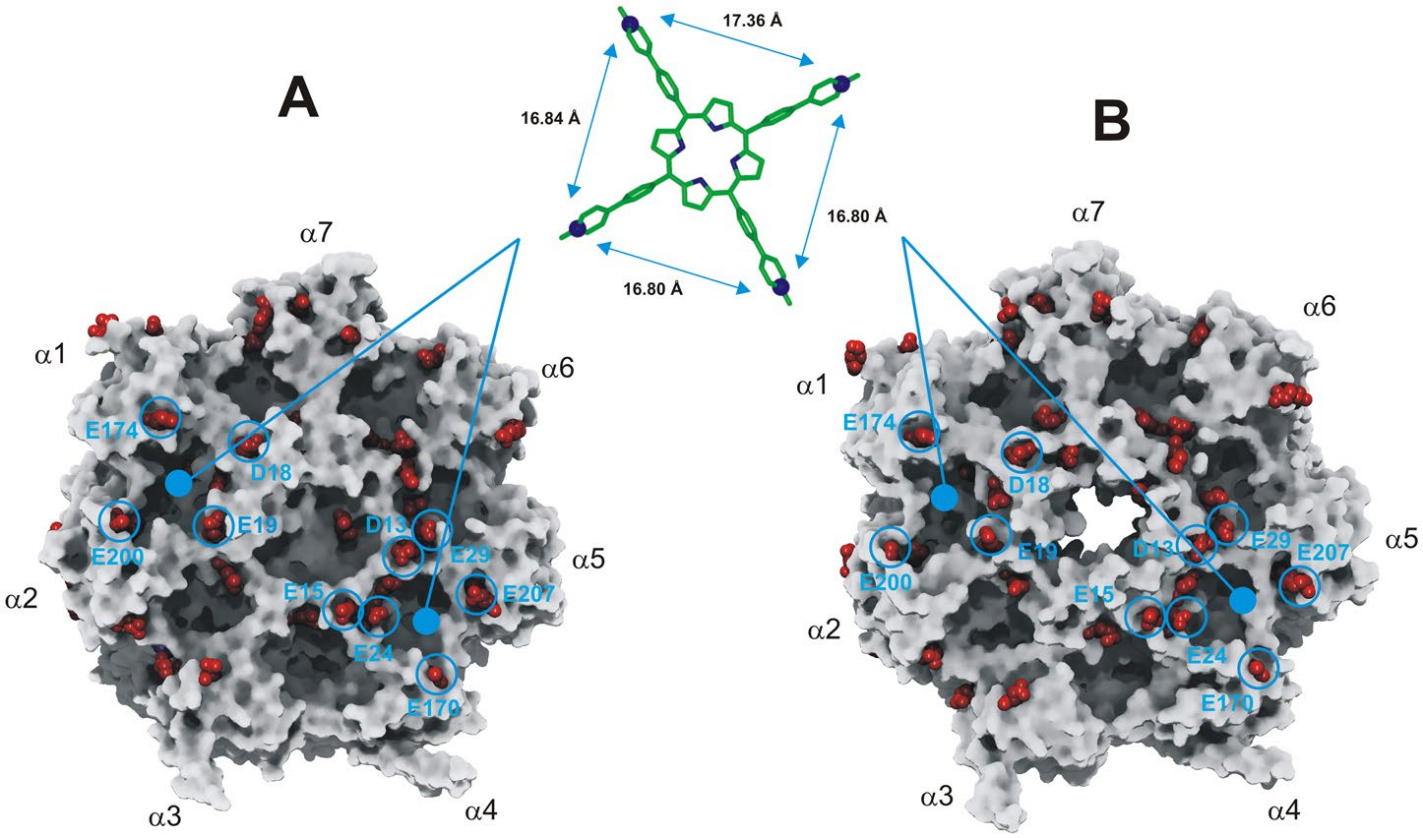
Putative binding sites of **2** on the α-ring surface of human 20S in the closed (**A**) and open (**B**) conformation. The putative interacting residues are labelled and circled in cyan. The ligand is displayed in stick and colored by atom type; hydrogens are omitted for sake of clarity. The inter-atomic distances among the pyridine nitrogen atoms (evidenced in CPK) are reported. The human 20S proteasome (top view) is displayed as Connolly surface and colored in gray except for negatively charged residues involved in ionic interactions with PA28, PA200, and 19S RPs which are displayed as CPK and colored in red.

The obtained results allowed us to map the spatial position of all negatively charged residues of human 20S involved in ionic interactions with PA28α, PA200, and 19S RPs. Interestingly, it resulted a regular arrangement of such residues on the α-ring surface (Fig. 4), disclosing a structural code accounting for the observed role played by ionic interactions in the allosteric regulation of proteasome catalytic activity^17,26^.

Importantly, our structural analysis identified the 20S negatively charged amino acids edging the α1-α2 and α4-α5 grooves, as putative binding sites for cationic porphyrin based ligands having a square planar conformation of the positively charged groups, such as in **1** (Fig. 4). However, as evidenced by the inter-atomic distances reported in the Supporting Information (Table S4), to allow the ionic interactions with the negatively charged residues of the protein, a larger distance between the positively charged groups of the ligand, with respect to that showed by **1** (11 Å), is required. This led us to identify the cationic porphyrin **2** (Fig. 1) as a putative ligand of such regulatory sites. Indeed, in the structure of **2** a phenyl ring is introduced into the spacer linking the pyridine substituent to the porphyrin core, thus resulting in a distance of about 17 Å between the adjacent charged groups (Fig. 4). However the presence of a larger cluster of negatively charged residues characterizing the α4-α5 groove, leading to several possible combinations of ionic interactions (i.e., binding modes), suggests that this is the most probable binding site for **2**. To test this hypothesis, **2** was subjected to dynamic docking studies in complex with human 20S.

### Docking Studies

**Figure 5:**
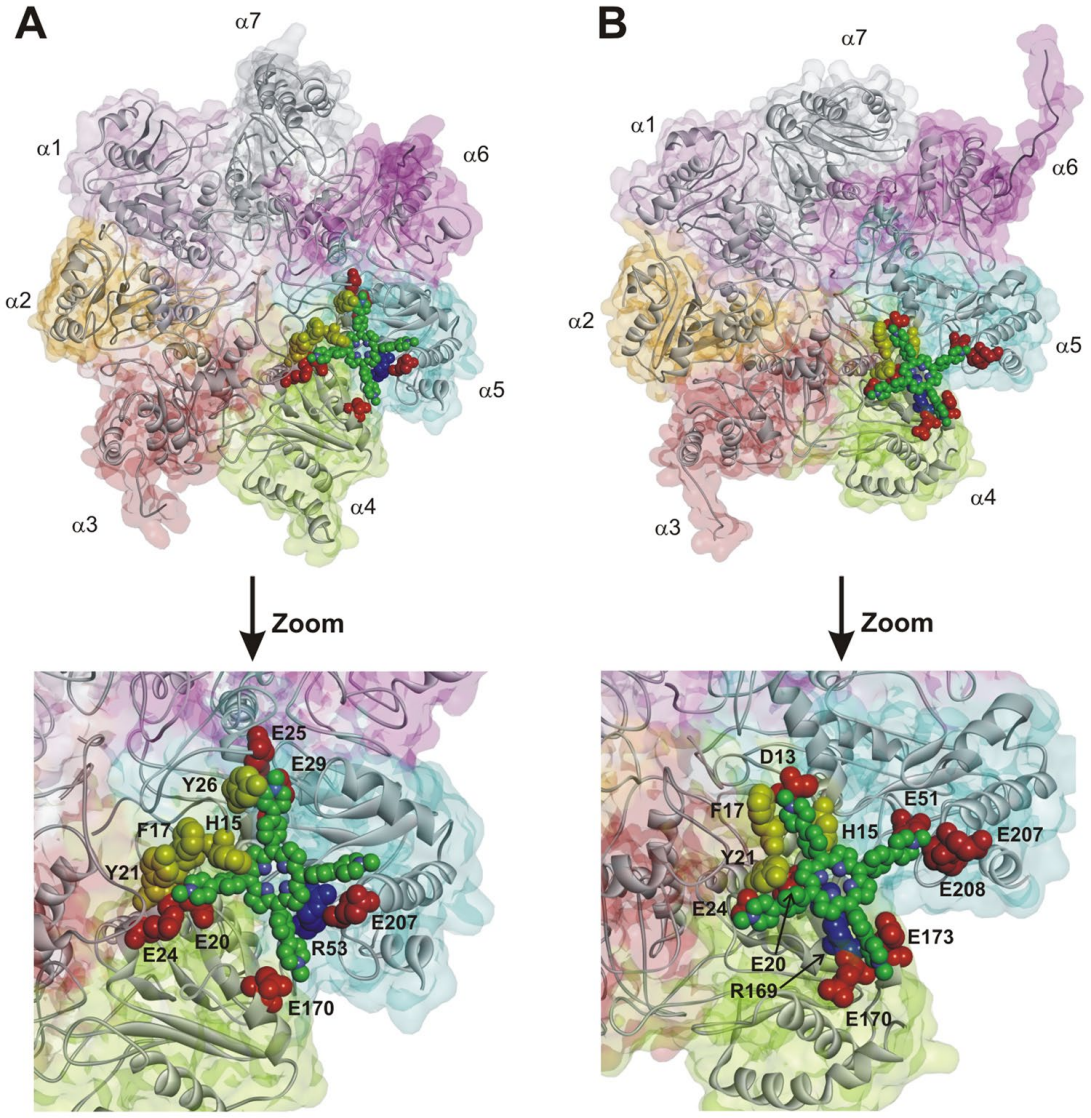
The 20S-**2** docked complexes obtained starting from the closed (**A**) or open (**B**) conformation of 20S. The α subunits are displayed as ribbons and colored in gray. **2** is displayed as CPK and colored by atom type (C: green; N: blue). The amino acid residues involved in ionic, cation-π and π-π interactions are displayed as CPK and colored in red (negatively charged residues), yellow (aromatic residues), and blue (positively charged residue). The protein van der Waals volume is displayed as transparent surface and colored by subunit type.

Firstly, the molecular models of the human 20S proteasome (closed and open conformation) were built and structurally optimized by molecular mechanics (MM) calculations (for details see SI). Then, in order to simulate the dynamics of ligand-protein recognition event, we applied an original docking procedure based on a Monte Carlo-Metropolis/simulated annealing (SA) calculation protocol^36–38^. Importantly, the whole structure was included in the calculation, allowing to fully explore possible *i*) ligand binding sites and/or modes and *ii*) ligand-induced protein large-scale conformational changes (for details see SI). Calculations were performed starting from both the closed and the open 20S conformation, giving rise to two sets of results (Figs. S4–S5). The resulting docked and annealed complexes were analyzed and the ligand-protein interaction energy was calculated (Group Based non-bond interactions method^39^) (Tables S5–S6, SI). The complexes with the best compromise between the non-bond interaction energies obtained by Monte Carlo and SA calculations (Complex 3, Table S5; Complex 9, Table S6), were selected as the structures representing the most probable binding modes (Fig. 5; Tables S8–S9 and S11, SI). The quality of the selected complexes was assessed by using Molprobity structure evaluator software^40^ (Table S7, SI).

The selected complexes show the ligand bound to the α4-α5 groove (Fig. 5). Moreover, not only the two selected complexes but also other energetically favored complexes, according to the calculated non-bond interaction energies reported in Tables S5 and S6 (i.e., Complex 1 and 2, Table S5; Complex 1,2,4,7,8 and 10, Table S6), show the ligand placed within the α4-α5 groove, thus confirming this latter as the most probable binding site for **2** on human 20S.

In the complex obtained when using the 20S closed conformation as starting structure, the cationic porphyrin **2** interacts with the α4-α5 groove by establishing: *i*) ionic interactions with six negatively charged residues (i.e., E20(α4), E24(α4), E170(α4), E25(α5), E29(α5), and E207(α5); all involved in the binding to RPs), *ii*) cation-π interactions with three aromatic residues (i.e., F17(α4), Y21(α4), and Y26(α5)) and one positively charged residue (i.e., R53(α5)), and *iii)* π-π interactions with a histidine residue (i.e., H15(α4)) (Fig. 5A and Table S8).

In this complex, the ligand-induced conformational changes are not limited to the α4-α5 groove, but include the interface with β5 extending to the S3 catalytic site pocket (see SI Fig. S6A). Importantly, such conformational changes were previously proved to be involved in the allosteric communication between the α ring surface and the catalytic centers (and vice versa) which regulates the dynamic equilibrium between open/closed conformation and, thus, the 20S catalytic activity^11,35^. However, unlike what observed in the shift from the closed to the open conformation^11^, the conformational changes induced by **2** led to the close up of the loop which defines the S3 pocket (i.e., aa20–30) toward the catalytic threonine (see Supplementary Fig. S6), in agreement with the observed capability of **2** to allosterically inhibit proteasome catalytic activity (described in the next paragraph). At the same time, the binding of **2** at the α4-α5 groove modified the conformation of the other α grooves, thus changing the spatial topology of the negatively charged residues (see Supplementary Fig. S7B *vs* S7A). Taking into account the below described cooperative binding of **2** to 20S, it is worth of note that five negatively charged residues at the α3-α4 groove, two of which interacting with RPs, result in a spatial arrangement able to interact with the four positively charged substituents of **2**, allowing the putative accommodation of a second ligand molecule (see Supplementary Fig. S7B and Table S10).

In the complex obtained when using the 20S open conformation as starting protein structure, **2** interacts with the α4-α5 groove establishing: *i*) ionic interactions with six residues also found to interact with RPs (i.e., D13(α4), E20(α4), E24(α4), E170(α4), E51(α5), and E207(α5)), *ii*) cation-π interactions with two aromatic residues (i.e., F17(α4) and Y21(α4)) and one positively charged residue (i.e., R169 (α4)), *iii*) a π-π interaction with H15(α4) (Fig. 5B, Table S11). Also in this case, upon the binding of **2** to the α4-α5 groove, conformational changes were induced to the entire α-ring surface, leading to the formation of an additional putative ligand binding site, which involves, this time, six negatively charged residues, five of which found to interact with RPs at the α5-α6 groove (see Supplementary Fig. S7D vs S7C; Tables S9 and S12). In addition, the α4 amino-terminal tail moved toward the substrate gate, interacting, at the same time, with the backbone carbonyl groups of residues G8 (α1), R3(α6), and N4 (α6) through charge assisted hydrogen bonds. This determined the positioning of α1, α4, and α6 amino-terminal tails at the substrate gate, leading to its steric occlusion. Conversely, the interaction of **2** with the open conformation of 20S induced minor conformational changes at the interface with the underlying β5 subunit (see Supplementary Figs S6B vs S6A). Accordingly, the positioning of the βH2 helix with respect the loop region (aa102–111), as well as the positioning of the loop region (aa20–30) with respect to the catalytic threonine, resulted similar to those present in the starting (open) structure (see Supplementary Fig. S6B).

Taken together, our docking results confirm that **2** may bind at the α4-α5 groove of human 20S, both in the closed and open conformation. It is noteworthy that the binding of **2** induces conformational changes favoring the accommodation of a second ligand molecule. For the same reason, it has to be reminded that our structural and bioinformatics analysis identified another putative binding site at the α1-α2 groove, which involves the negative residues responsible for the anchoring of RPs, totaling three putative binding sites for **2** on each α ring of the 20S CP.

### Potency and mechanism of inhibition of human 20S by 2

Figure S8 reports IC_50_ values of **2** (obtained through Eq. S1) for the three peptidase activities of the 20S proteasome (ChT-L, T-L, and PGPH-L). A comparison with the parent molecule **1** (i.e. H_2_T4) is further reported, showing that **2** inhibits to the same extent all three catalytic activities of the proteasome with IC_50_ values very similar and around 2 μM.

**Figure 6:**
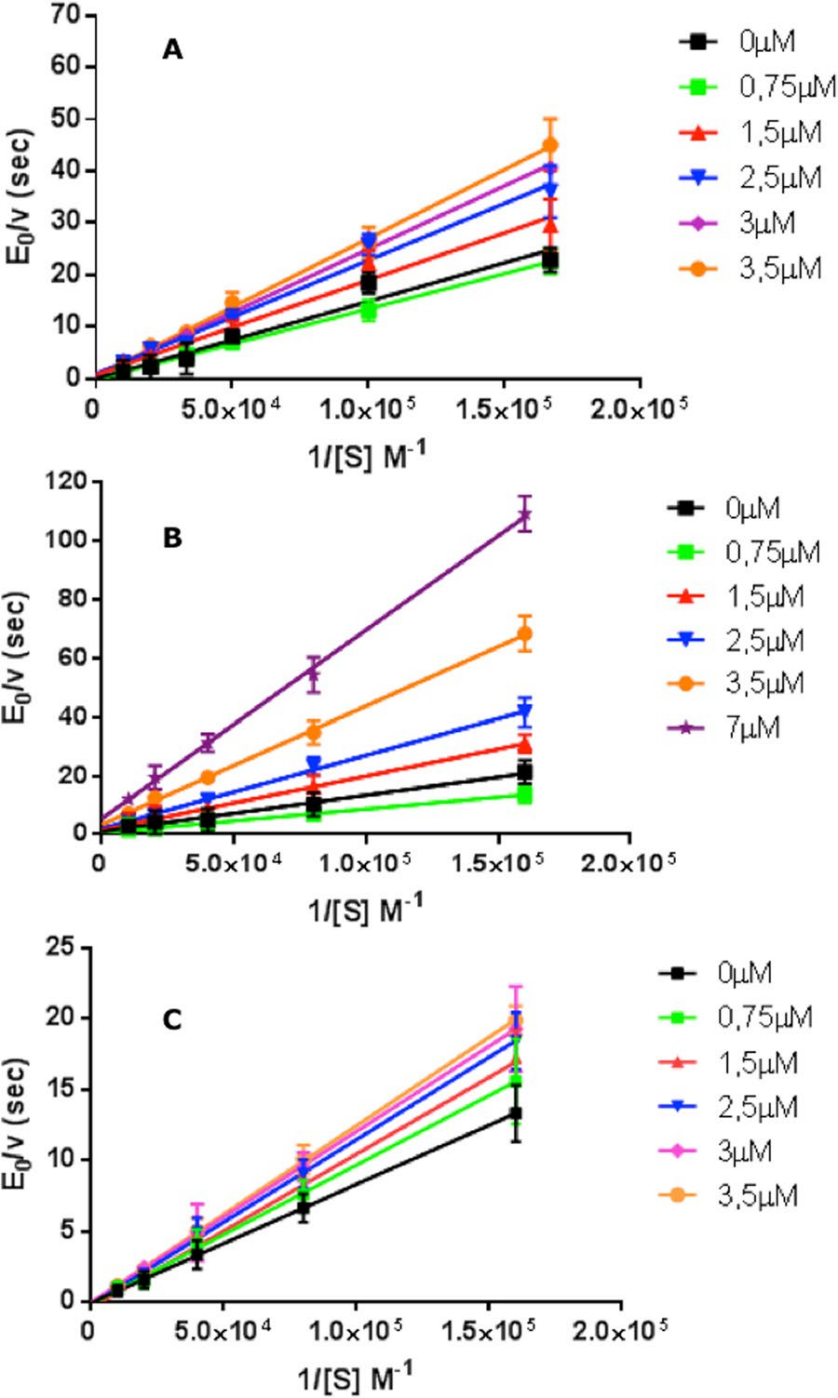
Double reciprocal Lineweaver-Burk plot of substrate Suc-LLVY-AMC enzymatic processing in the absence of inhibitor (black) and in the presence of increasing amounts of **2** (as indicated), for various concentrations of the substrate, by human 20S proteasome (*panel* A); by yeast 20S proteasome (*panel* B); by α-3ΔN yeast mutant 20S (*panel* C). Continuous lines are the non-linear least-squares fitting of data, employing Eqs. (S1, *panel* C) and (S3, *panels* A and B).

In order to investigate the inhibition mechanism of porphyrins, a steady-state kinetic analysis was carried out. The reaction rates were determined in the presence of increasing amounts of inhibitor (ranging between 6 μM and 100 µM); these results were plotted using the Lineweaver-Burk double reciprocal plot (Fig. 6 and Eq. S2), obtaining the values of catalytic parameters.

According to this analysis (see Eqs. (S3a-c) in SI) **2** displays a purely non-competitive inhibition mechanism with α ≈ 1 (see Eq. (S3c) and Fig. 6A) underlying the possibility that substrate binding does not affect porphyrin interaction. An almost pure non-competitive inhibitory mechanism can also be observed for the wild-type yeast 20S proteasome (see Fig. 6B), confirming that this porphyrin interacts in a closely similar way with human and yeast 20S proteasome. Interestingly, in the case of the yeast mutant α-3ΔN (where the first 9 amino acids at the *N*-terminus of the α3 chain have been removed, apparently locking the human 20S proteasome in the “open” conformation)^41,42^ the porphyrin acts as a competitive inhibitor (see Fig. 6C, Eq. (S3a) and See Supplementary Fig. S9).

**Figure 7:**
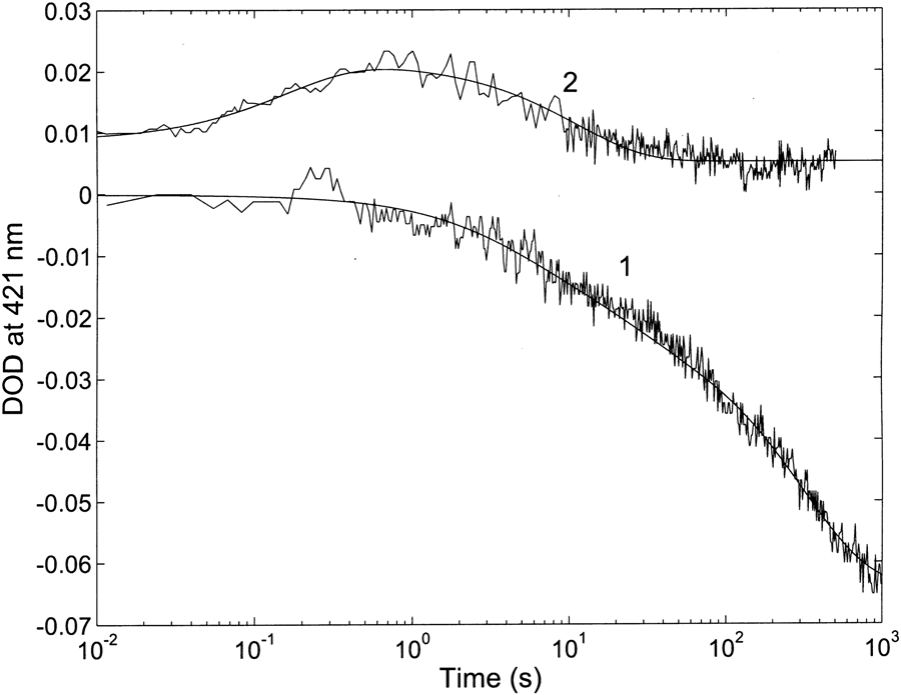
Comparison of the kinetic progress curves at 421 nm for the reaction of 1 nM of 20S proteasome with 5 μM of **2** and 5 μM of **1** (from ref.^28^) T = 37 °C. Continuous lines are the non-linear least-squares fitting of data according to Eq. (S8).

Figure 7 shows the UV stopped flow kinetic progress curve at 421 nm for the interaction between 5 μM of **2** with 1 nM of 20S proteasome as compared with reaction of 5 μM of **1**.

The reaction displays drastically different features, being characterized (in the case of **2**) by only two exponentials (they were three in the case of H_2_T4, see ref.^28^) and by a significantly (~10-fold) faster rates; unlike for **1**, there is no evidence, at least over the investigated porphyrin concentration range, of a very slow concentration-independent step. In addition, unlike for **1** (where for all binding steps a hypochromic behavior is observed, see Fig. 7), in the case of the binding of **2** to the 20S proteasome the first event is characterized by hyperchromism, followed by a second step displaying hypochromism.

The analysis of kinetic progress curves, employing Eq. (S8), allows to obtain the values of *k*_*obs*_ *a*t different porphyrin concentrations, which can be exploited, for a classical bimolecular behavior, to obtain the association and dissociation rate constants according to Eq. (1a)

1a $${k}_{obs}={k}_{on}\cdot [Porph]+{k}_{off}$$

**Figure 8:**
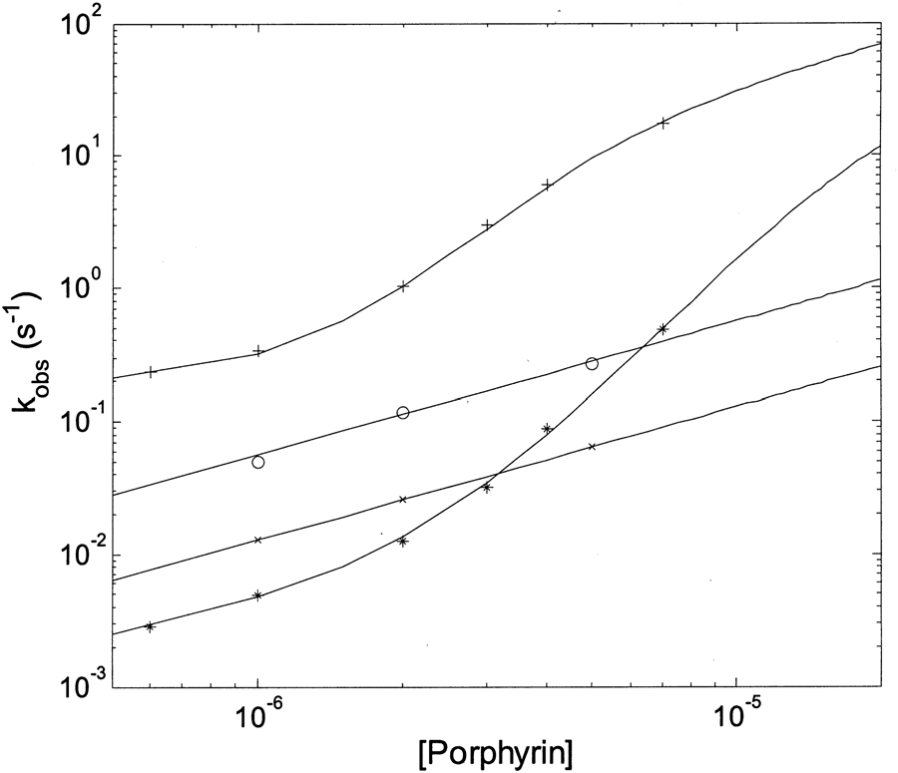
Porphyrin concentration dependence for the binding rate constants to human 20S proteasome at 37 °C of **1** (o, fast phase, x, slow phase, *see ref*.^28^) and **2** ( + , fast phase, *slow phase). Continuous lines have been obtained, employing Eq. (1a) for **1** and its modified form Eq. (1b) for **2**, employing parameters reported in Table S13.

However, the most peculiar result, obtained for **2**, is the concentration dependence of *k*_*obs*_, which shows a cooperative behavior (see Fig. 8). Thus, unlike for **1**, which follows a simple bimolecular behavior (see ref.^27^), **2** displays a cooperative concentration dependence both for the hyperchromic (fast) and the hypochromic (slow) process (Fig. 8). Therefore, the classical bimolecular behavior, as from Eq. (1a), has been modified accordingly:

1b $${k}_{obs}=({k}_{c}\cdot \frac{{P}_{c}}{{P}_{tot}}+{k}_{o}\cdot \frac{{P}_{o}}{{P}_{tot}})\cdot [Porph]+{k}_{off}$$

where *k*_c_ and *k*_o_ are the porphyrin binding rate constants to the 20S proteasome in the “closed” and in the “open” conformation, respectively, *k*_off_ is the dissociation rate constant of the porphyrin, *P*_c_ and *P*_o_ are the relative percentage of the two conformations (“closed” and “open”, respectively) as a function of porphyrin concentration, as from Eqs. (S4–S7) (see SI).

This behavior envisages the possibility that this porphyrin is a conformational modulator of the 20S proteasome, likely affecting the “open”-“closed” equilibrium. In this framework, the porphyrin concentration dependence of the observed rate constant (*i*.*e*., *k*_obs_) has been analyzed, implying that the two conformations are in equilibrium and are characterized by different porphyrin association rate constant (see SI).

Continuous lines for binding rate constants of **2** have been obtained according to Eq. (1b) and Eqs. (S4–S7) (see SI), employing n = 3, since lower values did not provide a slope steepness sufficient to account for the porphyrin concentration dependence (Fig. 8). The choice of n = 3 was also dictated by the structural evidence that both in the “open” and in the “closed” state the α-ring may undergo a porphyrin-linked conformational change, being able to accommodate up to three porphyrin molecules. Kinetic parameters for the binding of **2** to the human 20S proteasome are reported in Table S13, together with those observed for the reaction of **1** (see ref.^28^).

Such a formalism implies the existence of two clusters of sites for the porphyrin, each one hosting three molecules, displaying a different affinity according to whether the proteasome is in the “open” or in the “closed” conformation. Therefore, binding of porphyrins to either one of the two clusters shifts the conformational equilibrium toward either the “open” or the “closed” structure (see above).

From a closer view of parameters, reported in Table S13, it emerges that binding of porphyrin(s) to the first cluster induces a shift of the conformational equilibrium from the “closed” (which is prevalent in the absence of substrate) to the “open” structure (as indicated by the higher affinity for the “open” state, see Table S13); on the other hand, binding to the second cluster brings about the opposite shift (as indicated by the higher affinity for the “closed” state, see Table S13), so that fully porphyrin-bound proteasome comes back to a “closed” conformation (which is not necessarily the same as the initial one) characterized by a low enzymatic activity.

Therefore, the overall non-competitive inhibitory mechanism, displayed by **2**, finds its structural-functional basis on the shuttling, consequent to the sequential binding to the two clusters, between the two conformations. However, for the sake of clarity, it must be pointed out that this last phenomenon cannot be ascribed simply to the transition between the two conformations, likely reflecting instead the different environment in which porphyrins are bound to the 20S proteasome.

**Figure 9:**
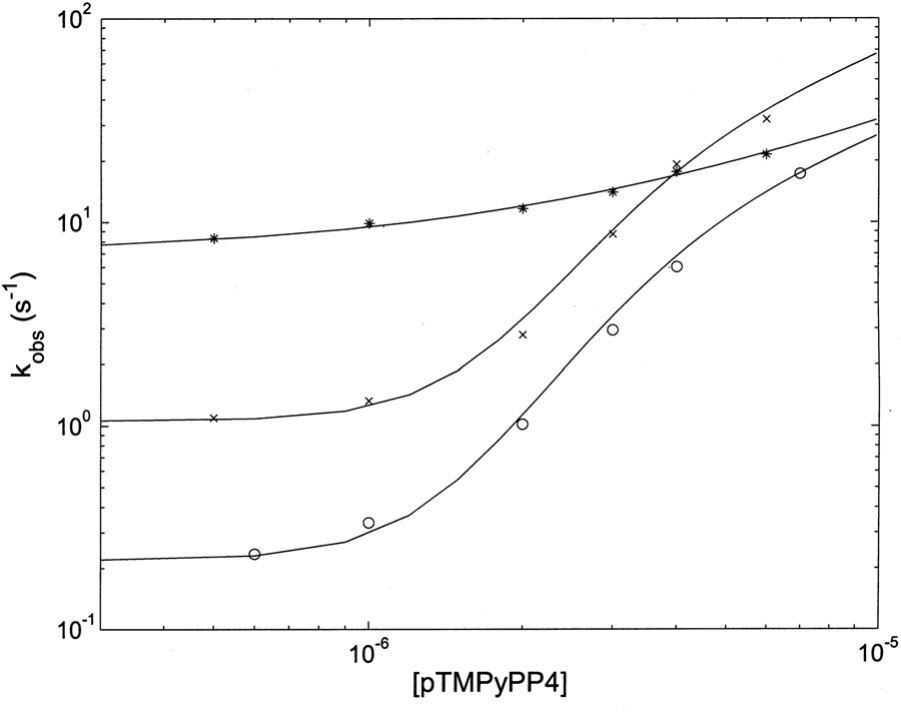
Dependence of *k*_*obs*_ for binding of **2** at 37 °C to the first cluster in human 20S proteasome (o), wild-type yeast 20S proteasome (x) and the α-3ΔN mutant of yeast 20S proteasome (*). Continuous lines have been obtained applying parameters reported in Table 1 to Eqs. (1a)(for the α-3ΔN mutant of yeast 20S proteasome) and (1b)(for the two wild-type proteasomes).

Figure 9 depicts a comparison between human 20S proteasome, yeast wild-type 20S proteasome and the yeast mutant α-3ΔN for the concentration dependence of **2** binding; parameters corresponding to the continuous lines are reported in Table 1. It is immediately evident that while both wild-types 20S proteasome (from human and from yeast) display a similar cooperative concentration dependence for rates (with slight differences, which concern only the rate constants for the “open” conformation, see Table 1) in the case of the α-3ΔN mutant binding of **2** shows a classical bimolecular concentration dependence, as already observed for **1** ^28^. This behavior mirrors what reported in Fig. 6, where the two wild-types display an allosteric non-competitive inhibitory effect upon **2** binding, while a simple competitive mechanism is detected for **2** binding to the α-3ΔN mutant (Fig. 6).

**Table 1:** Equilibrium and kinetic binding parameters of **2** to the first cluster of different 20S proteasomes (See Eqs. S4–S7).

	Human	Yeast	α-3ΔN
K_o_ (M^−1^)	1.8 × 10^7^	1.8 × 10^7^	3.6 × 10^5^
K_c_ (M^−1^)	7.0 × 10^3^	7.0 × 10^3^	—
k_o_ (M^−1^s^−1^)	2.8 × 10^6^	7.0 × 10^6^	2.5 × 10^6^
k_c_ (M^−1^s^−1^)	1.3 × 10^2^	1.3 × 10^2^	—
k_off_ (s^−1^)	0.22	1.06	7.0
L	4.0 × 10^−6^	4.0 × 10^−6^	—

This evidence is a clear demonstration that the cooperative concentration dependence is somehow related to the “open-closed” transition of the 20S proteasome, since it disappears when this transition is forbidden (as in the α-3ΔN mutant).

**Figure 10:**
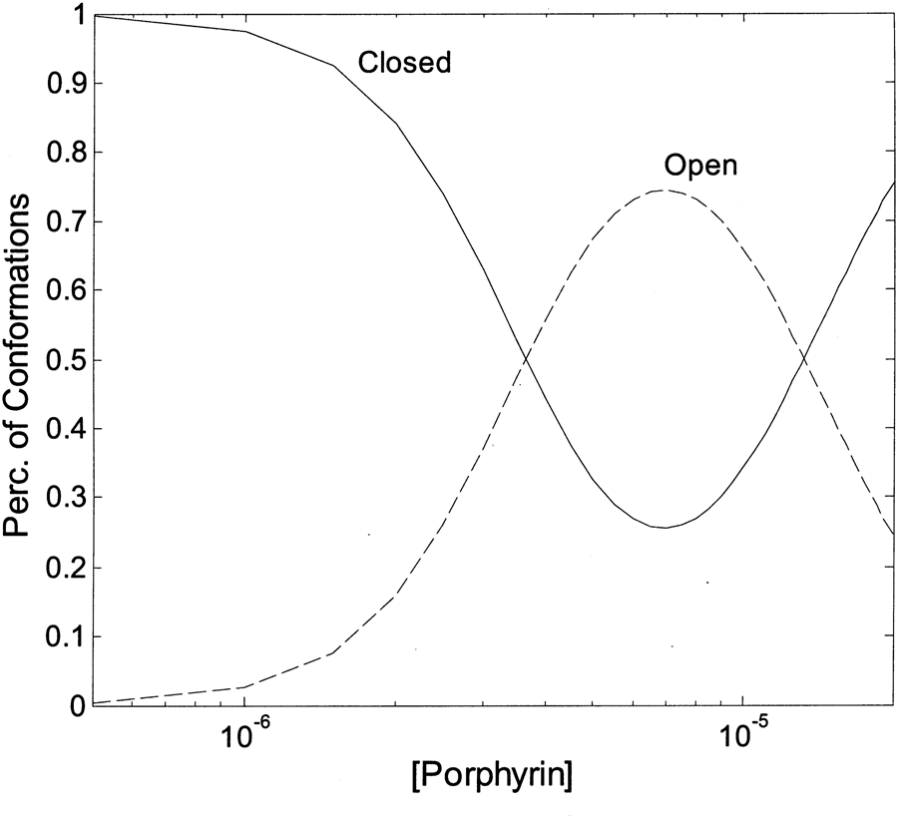
Percentage of “closed” and “open” conformation (as indicated) of human 20S proteasome as a function of porphyrin concentration, employing parameters reported in Table 1. Curves are theoretical ones for the “closed” (continuous curve) and “open” (dashed curve) as obtained by the application of Eqs. (S4) and (S5), respectively.

In conclusion, the overall picture emerging is the existence of (at least) two types of sites for **2** with somewhat different affinity constants. Each type displays a cluster of (at least) three interaction spots, functionally correlated in a concerted fashion, such that the degree of occupancy by the porphyrin displaces the conformational equilibrium toward one of the two main structures, *i*.*e*., “open” or “closed”. Computational results allowed to propose that porphyrin might bind at the α1-α2 and α4-α5 grooves (see Fig. 4), inducing a conformational change, which shapes a third binding site at the α5-α6 or α3-α4 groove, depending on initial conformational state of 20S (see Supplementary Fig. S7). This evidence gives an important structural basis to the observed cooperative interaction of the porphyrin (see Figs 7 and 8), allowing to sketch a proposed mechanism for this allosteric inhibitory modulation of the 20S proteasome. Initially, in the absence of porphyrins the equilibrium is in favor of the “closed” conformation and binding of porphyrins to the cluster of sites with higher affinity (named as “cluster 1”) shifts progressively the equilibrium toward the “open” conformation (see Fig. 10).

Interaction of the porphyrin with the second cluster of sites (named as “cluster 2”), characterized by a somewhat lower affinity, induces the conformational equilibrium to be displaced in favor of a “closed” structure (see Fig. 10), which is not necessarily the same which was predominant in the absence of porphyrins, and which will call “closed-bound” state. Obviously, the picture represented in Fig. 10 refers to the situation at equilibrium and it does not account for the different rates of porphyrin binding to the two cluster of sites, which is instead responsible for the behavior observed kinetically (see Figs 7 and 8). Thus, from the kinetic viewpoint as soon as some “open” structure is formed (upon porphyrin binding to the cluster 1), porphyrin binding to the cluster 2 may occur, inducing a backward shift toward the “closed-bound” state, even under conditions favoring at equilibrium the “open” conformation. For this reason, the simulation of the distribution of populations as a function of time cannot be undertaken without knowing the extinction coefficients of the intermediate partially bound clusters.

## Conclusions

The starting hypothesis of this work is that the spatial distribution of electrostatic charges, present at the surface of the α-rings, may guide the design of cationic porphyrins able to dynamically interfere with the proteasome activation mechanism. Therefore, on the basis of this information we have designed and synthesized **2**, a porphyrin wherein a phenyl spacer is incorporated between the aromatic core and each of the four protonated pyridine arms of the parent molecule **1**, allowing the interaction with the negatively charged residues at the α1-α2 and α4-α5 grooves in both the closed and the open 20S conformational states. Dynamic docking simulations evidenced that **2** might preferentially bind at the α4-α5 groove, inducing a conformational change, which shapes a third binding site, located at the α3-α4 groove (starting structure: closed 20S) or at the α5-α6 groove (starting structure: open 20S). Moreover, the overall conformational rearrangements induced by the binding of **2** to the α4-α5 groove reverberate onto the β5 subunit, inducing the closing of the S3 catalytic pocket (starting structure: closed 20S) or the steric occlusion of the gate by the α4 N-terminal tail (starting structure: open 20S). Proteasome activity experiments and UV stopped-flow binding kinetics performed in parallel on wild-type and the “fully open” α-3ΔN mutant 20S confirmed that **2** allosterically inhibits wild-type 20S by interfering with its gating equilibrium, while acting as a competitive inhibitor toward the α-3ΔN mutant. In addition, analysis of the kinetic data revealed the cooperative binding of at least three inhibitor molecules to two different clusters of interaction sites on human 20S, in full agreement with *in silico* results. Our findings corroborate the hypothesis that gating dynamics are involved in driving conformational movements of the catalytic β subunits and provide a framework for the design of increasingly sophisticated porphyrin-based allosteric proteasome inhibitors.

## Methods

The complete experimental description of: (i) chemical synthesis, (ii) proteasome activity assays, (iii) stopped-flow kinetic experiments, (iv) native gel electrophoresis, and (v) bioinformatics and molecular modeling studies is reported in the Supporting Information (SI).

## Electronic supplementary material

Supplementary information
