# Supplementary material for: Electrostatic Map Of Proteasome α-Rings Encodes The Design of Allosteric Porphyrin-Based Inhibitors Able To Affect 20S Conformation By Cooperative Binding
Source: Sci Rep. 2017 Dec 6;7:17098. doi: 10.1038/s41598-017-17008-7 (PMC5719074; doi:10.1038/s41598-017-17008-7)
Supplement: Supplementary file 1 — Supplementary information [file 41598_2017_17008_MOESM1_ESM.pdf]

SUPPORTING INFORMATION

Electrostatic Map Of Proteasome α-Rings Encodes The Design of Allosteric Porphyrin-Based Inhibitors Able To Affect 20S Conformation By Cooperative Binding.

Antonio Di Dato,[1] Alessandra Cunsolo,[2] Marco Persico,[1] Anna Maria Santoro,[3] Alessandro D'Urso,[2] Danilo Milardi,[3] Roberto Purrello,[2*] Manuela Stefanelli,[4] Roberto Paolesse,[4*] Grazia R. Tundo,[5] Diego Sbardella,[5] Caterina Fattorusso,[1*] Massimo Coletta[5*]

[1] Dipartimento di Farmacia Università di Napoli "Federico II", Via D. Montesano, 49 I-80131 Napoli, Italy.
[2] Dipartimento di Scienze Chimiche, Università degli Studi di Catania, Viale A. Doria 6, 95125 Catania, Italy.
[3] Istituto di Biostrutture e Bioimmagini - CNR sede secondaria di Catania, Via P. Gaifami,9- 95126 Catania, Italy
[4] Dipartimento di Scienze e Tecnologie Chimiche, Università di Roma Tor Vergata-Via della Ricerca Scientifica -00133Roma, Italy.
[5] Dipartimento di Scienze Cliniche e Medicina Traslazionale, Università di Roma Tor Vergata, Via Montpellier 1, I-00133 Roma, Italy.

Table of Contents:

1. Title, Affiliations, Table of Contents **S1**

2. Material and Methods: chemicals, synthesis, proteasome activity assay, stopped-flow kinetic experiments, native gel electrophoresis, and molecular modeling **S2-S11**

3. Synthesis (Figure S1 and S2) **S12-S13**

4. Molecular modeling studies (Figures S3-S8) **S14-S18**

5. Pharmacological and biochemical studies (Figure S9-S11) **S19-S21**

6. Molecular modeling studies (Tables S1-S12) **S22-S33**

7. Kinetic studies (Table S13) S34

8. References **S35**

MATERIALS AND METHODS

Chemicals. Purified human 20S proteasome and fluorogenic ChT-L (Suc-LLVY-AMC) and CP-L fluorogenic substrates (Z-LLE-AMC) were purchased from Boston Biochem (Cambridge, MA, USA). The T-L (Ac-RLR-AMC) substrate was purchased from Enzo Life Sciences (Farmingdale, NY, USA).

The meso-tetrakis(4-N-methylpyridyl) porphyrin (H_2T4), was purchased from Midcentury. The water soluble porphyrin pTMPyPP4 (**2**) was obtained in two synthetic steps and the detailed

synthetic strategy is reported. All chemicals were obtained from commercial sources (Aldrich, Merck or Fluka) and used as received. Silica gel 60 (70-230 mesh, Sigma Aldrich), neutral or basic alumina (grade III Brockmann) were used for column chromatography. ^{1}H NMR spectra were recorded on a Bruker AV300 (300 MHz) spectrometer. Chemical shifts are given in ppm relative to 3.31 ppm for MeOD, or 2.62 ppm for DMSO-d_6. UV/Vis spectra were measured on a Cary 50 spectrophotometer.

Synthesis of porphyrin derivatives 2* and 2. The water soluble porphyrin **2** was obtained in two synthetic steps, as outlined in Scheme 1. Following the Adler-Longo's methodology, we reacted equimolar amounts of 4-(4-pyridinyl)benzencarbaldehyde and pyrrole in boiling acetic acid, obtaining porphyrin **2*** in a 22% yield after chromatographic procedures. The cationic porphyrin **2** was obtained after exhaustive N-methylation of **2*** by a large excess of iodomethane in dry DMF in quantitative yield, by crystallization.

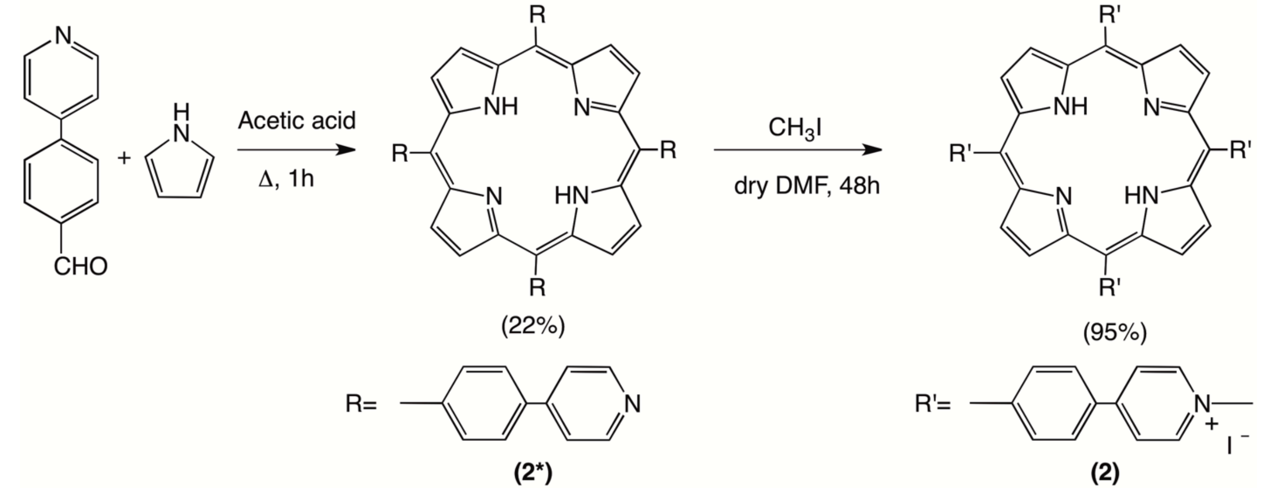

Scheme 1. Preparation of porphyrin derivatives **2*** and **2**.

Synthesis of 5,10,15,20-Tetrakis-[4-(4-Pyridinyl)-phenyl]porphyrin *(2*)*. Pyrrole (153 µL, 2.21 mmol) and 4-(4-pyridinyl)benzencarbaldehyde (404 mg, 2.21 mmol) were dissolved in acetic acid (20 mL) and the mixture was stirred at reflux for 1 hour. The solvent was removed under reduced pressure and the reaction crude was subjected to a first chromatographic purification by basic alumina, using $CHCl_3$ as eluant. All the fractions containing porphyrin were collected, concentrated and then further purified by a silica gel column, eluting with a chloroform/ethanol 6% solvent mixture. Porphyrin (**2***) was obtained as a purple powder (114 mg, 22% yield) by crystallization from methanol/water. Mp > 300 °C. UV-vis (THF): λ_{max}, nm (log ε) 421 (4.89), 514 (4.08), 551 (3.97), 593 (3.72), 651 (3.58). ^{1}H NMR (300 MHz, MeOD-TFA): δ = 9.14 (d, 8 H, J = 6.60 Hz, phenyl), 9.00 (d+s, 8 H, pyridinyl + 8 H, *β*-pyrrole), 8.87 (d, 8 H, J = 6.60 Hz, phenyl), 8.78 (d, 8 H, J = 8.10 Hz, pyridinyl). Anal. calcd for $C_{64}H_{42}N_8$: C, 83.27; H, 4.59; N, 12.14%. Found: C, 83.19; H, 4.47; N, 12.07%.

Synthesis of 5,10,15,20-Tetrakis-[4-(4-(N-Methyl)-pyridinium)phenyl]porphyrin-tetraiodide salt. *(2)*. Porphyrin (**2***) (110 mg, 0.12 mmol) was dissolved in 20 mL of anhydrous DMF, and CH_3I (1.5 mL, 24 mmol) was added. The mixture was stirred at room temperature in the dark, under nitrogen for 48 hours. The reaction mixture was then reduced to a small volume and then crystallized by adding equal volume of diethyl ether. The precipitate was washed several times with

diethyl ether, then with hexane to yield the desired N-alkylated porphyrin (**2**) as a purple solid (168 mg, 95% yield). Mp > 300 °C. UV-vis (DMSO): λ_{max}, nm (log ε): 428 (5.30), 520 (4.28), 555 (4.13), 594 (3.89) 644 (3.85). ^{1}H NMR (300 MHz, DMSO-d_6): δ = 9.19 (d, 8 H, J = 6.60 Hz, phenyl), 8.97 (s, 8 H, *β*-pyrrole), 8.85 (d, 8 H, J = 6.60 Hz, pyridinium), 8.55 (m, 16 H, phenyl pyridinyl), 4.45 (s, 12 H, N-CH_3), -2.85 (s, 2H, inner N*H*). Anal. calcd for $C_{68}H_{54}I_4N_8$: C, 51.56; H, 3.65; N, 7.52%. Found: C, 51.49; H, 3.60; N, 7.56%.

Proteasome activity assay. 2 nM of 20S proteasome was incubated with increasing concentrations of inhibitor (ranging from 0.5 to 3 µM) for 30 min at 37°C in the assay buffer (50 mM HEPES, 1mM EDTA, pH7.5) and, subsequently, 100 µM of AMC-labeled substrate peptide was added Latent Core Particles (CP). Proteasome activity was monitored by measuring the AMC fluorescence at 440 nm (excitation at 360nm) for 20 min, using a fluorescence plate reader (Multiskan, Thermo) in a 384 multiwell black plate. A minimum of three replicates were performed for each data point. Fluorescent substrate cleavage by the 20S proteasome was linear during this incubation time frame. Data are expressed as normalized percentages of residual activity considering the slope of the control (fluorogenic peptide/proteasome in the absence of inhibitors) as 100% of proteasome activity. Dose-response plots of the residual proteasome activity in the presence of increasing concentration of inhibitor provides a quantitative estimate of its potency. The IC_{50} is defined as the concentration of the inhibitor which causes 50% reduction of activity and it is thus calculated from the x-axis value on a semilog scale of the dose−response plot occurring at a fractional activity of 50%. The estimation of the IC_{50} is based on a nonlinear fit with a sigmoidal functions expressed by the equation:

$$v(\%) = \frac{100}{1+10^{([I]-LogIC_{50})}} \qquad \text{(S1)}$$

The midpoint of this sigmoidal function occurs at a fractional velocity value of 50%, corresponding to half inhibition of the target enzyme.

Data analysis. To visualize the effect of the inhibitor concentration the conversion rate (normalized for the enzyme concentration, v/[E_{tot}]) was measured as a function of substrate concentration (between 6 µM and 100 µM) for each inhibitor concentration (I) and analyzed as a double-reciprocal Lineweaver-Burk plot

$$\frac{[E_0]}{v} = \frac{K_m}{k_{cat}} \cdot \frac{1}{[S]} + \frac{1}{k_{cat}} \qquad \text{(S2)}$$

where [E_0] is the total enzyme concentration, v (moles of substrate / (volume x time)) is the enzymatic rate, [S] is the substrate concentration, k_{cat} is the speed of the rate-limiting step and K_m is the Michaelis-Menten constant, corresponding to the enzyme:substrate affinity. In order to distinguish among the different inhibition mechanisms, data were fitted according to Eqs. (S1-S3). All curve fitting and statistical analysis were carried out using the Non Linear Fitting Tool (NLFit) in Origin7. The parametric data fitting was based on nonlinear regression and the method of least squares. Model discrimination and choice was based on the goodness of fit. The goodness of fit was evaluated by visual examination of the fitted curves, 95% confidence bounds for the fitted coefficients and statistical analysis for determining the square of the multiple correlation coefficient (R^2).

Data analysis. Data analysis of enzymatic assays has been accomplished according to the non-linear least-squares fitting of Lineweaver-Burk equations assuming either a competitive inhibition equation:

$$\frac{[E_{tot}]}{v} = \frac{K_m \cdot (1 + \frac{[I]}{K_I})}{k_{cat}} \cdot \frac{1}{[S]} + \frac{1}{k_{cat}} \qquad \text{(S3a)}$$

or an un-competitive inhibition equation:

$$\frac{[E_{tot}]}{v} = \frac{K_m}{k_{cat}} \cdot \frac{1}{[S]} + \frac{(1 + \frac{[I]}{K_I})}{k_{cat}} \qquad \text{(S3b)}$$

or else a mixed inhibition equation:

$$\frac{[E_{tot}]}{v} = \frac{K_m \cdot (1 + \frac{[I]}{K_I})}{k_{cat}} \cdot \frac{1}{[S]} + \frac{(1 + \frac{[I]}{\alpha \cdot K_I})}{k_{cat}} \qquad \text{(S3c)}$$

in which k_{cat} is the rate-limiting step of the reaction, K_i is the dissociation constant for inhibitor I, K_M is the Michaelis-Menten constant for substrate binding, α is an interaction parameter (reflecting the difference of inhibitor affinity between the free enzyme E and the enzyme-substrate ES complex), [S] and [I] are the substrate and inhibitor concentrations, respectively.

The three different mechanisms reflect three different ways by which an inhibitor acts on the enzyme activity, namely (*i*) a competitive inhibitor interacts only with the free enzyme, (*ii*) an un-competitive inhibitor binds only to the enzyme-substrate complex, and (*iii*) a mixed inhibitor reacts with both the free enzyme and the enzyme-substrate complex, the α parameter indicating the different affinity for the two forms. When α is very large, binding of inhibitor impairs binding of the substrate and the mixed-model becomes identical to competitive inhibition. When α = 1 the mixed model becomes a purely non-competitive model.

Cooperativity for Porphyrin Binding. In the presence of (at least) two states (*i.e.*, "open" and "closed" in equilibrium) in 20S proteasome, the observed cooperative binding by **2** (see Fig. **7**) can be described by Eq. **(1b)** , where P_c

$$P_c = \frac{(1 + K_c \cdot [Porph])^n}{P_{tot}} \qquad \text{(S4)}$$

corresponds to the population of "closed" 20S as a function of porphyrin concentration (with K_c being the porphyrin equilibrium binding constant for this conformation) and P_o

$$P_o = \frac{L \cdot (1 + K_o \cdot [Porph])^n}{P_{tot}} \qquad \text{(S5)}$$

Refers to the population of "open" 20S as a function of porphyrin concentration, K_o being the equilibrium porphyrin binding constant and L is the conformational equilibrium between the two states in the absence of porphyrins, as from Eq. (S6)

$$L = \frac{[E_o]}{[E_c]} \qquad \text{(S6)}$$

Therefore, the population distribution in terms of bound porphyrins to the 20S proteasome in the two conformations as a function of porphyrin concentration can be described by Eq. (S7)

$$P_{tot} = (1 + K_c \cdot [Porph])^n + L \cdot (1 + K_o \cdot [Porph])^n \qquad \text{(S7)}$$

where "*n*" is the total number of porphyrins which can bind to that cluster in a concerted way. It is important to remark that application of these thermodynamic equations to the kinetic behaviour implies that the rate of the "open"-"closed" conformational transition is faster than the bimolecular

porphyrin binding. Employing values of K_c, K_o and L, reported in Table S13, it is possible to describe the dependence of k_{obs} on porphyrin 2 concentration according to Eq. (1b) (see Figs. 8 and 9), applying values of k_c and k_o, reported in Table S13.

Stopped-flow kinetic experiments. Experiments have been carried out by rapid-mixing experiments using the SX18.MV stopped-flow apparatus (Applied Photophysics, Salisbury, UK) equipped with a diode array for spectra acquisition over a 1 ms time range. Kinetics has been investigated by mixing 1 nM 20S proteasome with different concentrations of porphyrin(s) and following optical density changes as a function of time. Kinetic progress curves at selected wavelengths were analyzed according to the following equation:

$$OD_{obs} = OD_0 \pm \sum_{i=1}^{i=n} DOD_i \cdot \exp(-{}^{i}k_{obs} \cdot t) \qquad \text{(S8)}$$

where OD_{obs} is the observed optical density at a selected wavelength and at a given time interval, OD_0 is the optical density at t = 0, n is the number of exponentials, DOD_i is the optical density change associated to the exponential i, ${}^{i}k_{obs}$ is the observed rate constant of the exponential, and t is the time.

Native Gel Electrophoresis. The native gel analysis was performed according to the procedure described elsewhere.[3] 1 µg of the purified 20S proteasome was resolved under native conditions after a pre-incubation with the indicated concentration of 2, ranging from 0.3 to 10 µM in 25 mM Tris-HCl (pH 7.5). Thereafter, the particles were probed with 100 µm Suc-LLVY-AMC. It is worth recalling that while at 1 and 10 µM of 2 we observed an inhibition of the proteolytic activity of the 20S in the fluorimetric approach, these concentrations appear to be linked to an activation effect if the extent of degradation of the fluorogenic peptide is compared to that of the 20S in the absence of **2** by native gel. This behaviour is likely attributable to the highly different experimental conditions between the fluorimetric assays and the native gel.
The hypothesis concerning the existence of two clusters of sites on the 20S proteasome finds further correspondence with the data obtained by native gel electrophoresis. (see Figure S10), where a significant activation of the proteolytic activity of the purified 20S proteasome on the Suc-LLVY-AMC substrates was seen at 0.3 µM **2** followed by a progressive decrease of the proteolytic activity at higher *p*-TMPyPP4 concentrations.

Molecular modeling. Molecular modeling calculations were performed on SGI Origin 200 8XR12000 and E4 Server Twin 2 x Dual Xeon 5520, equipped with two nodes. Each node: 2 x Intel Xeon QuadCore E5520, 2,26Ghz, 36 GB RAM. The molecular modeling graphics were carried out on a personal computer equipped with Intel(R) Core(TM) i7-4790 processor and SGI Octane 2 workstations.
Structural and bioinformatics analysis of 20S proteasome. The experimentally determined structures of i) human 20S proteasome in complex with its regulatory particle (RP) (i.e., 19S) (PDB IDs: 5L4G (Cryo-EM; resolution 3.9 Å), 5GJQ (Cryo-EM; resolution 4.35 Å), 5GJR (Cryo-EM; resolution 3.5 Å), 5T0C (Cryo-EM; resolution 3.88 Å), 5T0G (Cryo-EM; resolution 4.4 Å), 5T0H (Cryo-EM; resolution 6.8 Å), 5T0I (Cryo-EM; resolution 8.0 Å), and 5T0J (Cryo-EM; resolution 8.0 Å)) and ii) *Saccharomyces cerevisiae* 20S proteasome in complex with its RPs (i.e., PA26, 19S and Blm10) and regulatory peptides (i.e., PR-VI and Blm-Pep) (PDB IDs: 1Z7Q (X-ray diffraction; resolution 3.22 Å), 4V70 (X-ray diffraction; resolution 3.01 Å), 4CR2 (Cryo-EM; resolution 7.7 Å),

4CR3 (Cryo-EM; resolution 9.3 Å), 4CR4 (Cryo-EM; resolution 8.8 Å), 3JCO (Cryo-EM; resolution 4.8 Å), 3JCP (Cryo-EM; resolution 4.6 Å)), 4X6Z (X-ray diffraction; resolution 2.7 Å), and 4ZZG (X-ray diffraction; resolution 3.0 Å)) were downloaded from the Protein Data Bank (PDB; http://www.rcsb.org/pdb/). Hydrogens were added to all the PDB structures assuming a pH of 7.2. These structures were analyzed using Biopolymer and Homology modules of Insight 2005 (Accelrys, San Diego). In particular, in order to analyze ionic interactions between 20S proteasome and its modulators, for each co-crystallized complex a subset around the 20S charged residues including all RP amino acids having at least one atom within a 5 Å radius from any given 20S charged residue was defined. Moreover, in the analysis of yeast 20S proteasome in complex with the RPs PA26 and Blm10, in order to verify if such interactions are conserved between human 20S and its modulators, we considered both the X-ray structures of yeast 20S and our homology models of human 20S (see following paragraph) superimposed on yeast 20S in complex with activators and we also performed: i) sequence alignments between yeast and human 20S proteasome subunits as well as proteasome RPs using PROMALS3D server (http://prodata.swmed.edu/promals3d/promals3d.php)[4] and ii) a structural superimposition, where it was possible, between human (PA28, PDB ID:1AVO; X-ray diffraction; resolution 2.8 Å) and non human RPs (PA26, PDB ID: 1ZTQ). Then, the new identified charged residues were introduced in the yeast RPs (Replace Residue command; Biopolymer module; Insight2005) and the putative ionic interactions were analyzed using different charged residue side chain rotamers (Manual_Rotamer command; Biopolymer module; Insight2005). Finally, all charged amino acids involved in these interactions were mapped on human 20S proteasome, in the closed and open conformation.

Modeling of human 20S proteasome. Since all human 20S proteasome experimentally determined structures are incomplete, the molecular models of human 20S proteasome in the closed and open conformation were built. All subsequent structural calculations were performed using the CVFF force field. [5]

Modeling of human 20S proteasome in the closed conformation. The molecular model of human 20S proteasome in the closed conformation was built starting from the experimentally determined structure of human 20S proteasome (PDB ID: 4R3O), which lacks the N-terminal regions (α1: aa1; α2: aa1; α3: aa1; α4: aa1; α5: aa1-7; α6: aa1-3; α7: aa1), C-terminal regions (α1: aa246; α3: aa252-261; α4: aa245-248; α6: aa242-263; α7: aa247-255; β1: aa203-205; β2: aa221-234; β4: aa200-201; β5: aa202-204; β7: aa218-219), and the side chains of several residues (α1: aa2, 3, 45, 46, 55, 59, 62, 63, 146, 181,186-188, 196, 223, and 245; α2: aa4, 18, 36, 52-55, 60, 139-141, 143, 163, 176-178, 184, 193, 195, 196, 199, 200, 202, 226, 227, 230, and 233; α3: aa13, 37, 51, 52, 54, 177, 184, 194, 197, 201, 205, 210, 222, 229-231, 250, and 251; α4: aa27, 40, 42, 43, 46-50, 53, 54, 56, 61, 68, 84, 136-138, 141, 157, 163, 166, 169, 170, 174, 181-183, 185, 187-189, 192, 193, 195-199, 204, 207, 208, 211-213, 216, 218, 223-227, 229, and 231-244; α5: aa8, 86, 187, 192, 208, 209, and 231; α6: aa208, 217, 218, 237, 238, and 241; α7: aa109, 143, 203, 205, 207, 223, and 245; β1: aa201-202; β2: aa9, 180, 206, and 214; β3: aa47; β4: aa41, 95, 109, 185, 198, and 199; β5: aa106, 150, and 185; β6: aa45, 161, 166, and 200; β7: aa44, 156, 170, 195, and 216). The sequence of 4R3O was aligned with the sequences of human 20S proteasome downloaded from the UniProtKB/Swiss-Prot Data Bank (http://www.uniprot.org; entry P60900 (α1); P25787 (α2); P25789 (α3); O14818 (α4); P28066 (α5); P25786 (α6); P25788 (α7); P28072 (β1); Q99436 (β2); P49720 (β3); P49721 (β4); P28074 (β5); P20618 (β6), and P28070 (β7)) by using the Multiple_Alignment algorithm (Homology module, Accelrys, San Diego). Subsequently, the secondary structural prediction of human 20S proteasome was performed using the Structure Prediction and Sequence Analysis server PredictProtein (http://www.predictprotein.org/). The coordinates of the structurally conserved regions (α1: aa2-245; α2: aa2-234; α3: aa2-251; α4: aa2-244; α5: aa8-241; α6: aa4-241; α7: aa2-246; β1: aa1-202; β2: aa1-220; β3: aa1-204; β4: aa1-199; β5: aa1-201; β6: aa1-213; β3: aa1-217) were accordingly assigned by the Structurally Conserved Regions (SCRs)-AssignCoords procedure

(Homology Module, Insight 2005) using 4R3O as template structure. On the other hand, the coordinates of the lacking N-terminal and C-terminal amino acids were assigned using the EndRepair command (Homology Module, Insight 2005). The obtained homology model was completed inserting the missing residue side chains by using the Replace command (Biopolymer module, Accelrys, San Diego), and the water molecules of experimentally determined structure human 20S proteasome (PDB ID: 4R69) through the UnMerge and Merge commands (Biopolymer module, Accelrys, San Diego). The obtained homology model was then subjected to a full energy minimization within Insight 2005 Discover_3 module (Steepest Descent algorithm, maximum RMS derivative = 0.5 kcal/Å; ε = 80*r). During the minimization, only the whole disordered N- and C-terminals and the SCRs side chains were left free to move, whereas the SCRs backbone were fixed to avoid unrealistic results. Each step of refining procedure was followed by a structural check by using the Struct_Check command of the ProStat pulldown in the Homology module to verify the correctness of the geometry optimization procedure before moving to the next step. Checks included φ, ψ, χ1, χ2, χ3, and ω dihedral angles, Cα virtual torsions, and Kabsch and Sander main chain H-bond energy evaluation. The quality of the resulting complexes was then checked using Molprobity structure evaluator software [6] and compared to that of the reference PDB structure. The obtained homology model was used for successive dynamic docking studies.

Modeling of human 20S proteasome in the open conformation. According to the reference structure (PDB ID: 5T0J), it must be highlighted that the molecular model of 20S in the open conformation was built considering only two packed rings (i.e., one α subunit ring and one β subunit ring). The molecular model of human 20S proteasome in the open conformation was built starting from the experimentally determined structure of human 20S proteasome (PDB ID: 5T0J), which lacks the N-terminal regions (α1: aa1-4; α2: aa1-2; α3: aa1; α4: aa1-2; α5: aa1-8; α6: aa1-3; α7: aa1-5), C-terminal regions (α1: aa245-246; α3: aa252-261; α4: aa241-248; α6: aa242-263; α7: aa246-255; β1: aa192-205; β2: aa221-234; β4: aa200-201; β5: aa202-204; β7: aa216-219), one loop region (α5: aa128-133), and the side chains of several residues (α1: aa45, 46, 55, 59, 63, 146, 181, 186-188, 196, and 223; α2: aa18, 36, 52-55, 60, 139-141, 143, 163, 176-178, 184, 193, 195, 196, 199, 200, 202, 226, 227, 230, and 233; α3: aa13, 37, 51, 52, 54, 177, 184, 194, 197, 201, 205, 210, 222, 229-231, and 250-251; α4: aa27, 40, 42, 46-50, 53, 136, 138, 141, 157, 163, 166, 169, 170, 174, 181-183, 185, 187, 189, 192-193, 195-199, 204, 207, 208, 212-213, 216, 218, 223, 225-227, 229, 232-237, and 239-240; α5: aa8, 86, 187, 192, 208-209, and 231; α6: aa208, 217-218, 237-238, and 241; α7: aa109, 143, 203, 205, 207, and 223; β2: aa9, 180, 206, and 214; β3: aa47; β4: aa41, 95, 109, 185, 198, and 199; β5: aa106, 150, and 185; β6: aa45, 161, 166, and 200; β7: aa44, 156, and 195).

The sequence of 5T0J was aligned with the sequences of human 20S proteasome downloaded from the UniProtKB/Swiss-Prot Data Bank (http://www.uniprot.org; entry P60900 (α1); P25787 (α2); P25789 (α3); O14818 (α4); P28066 (α5); P25786 (α6); P25788 (α7); P28072 (β1); Q99436 (β2); P49720 (β3); P49721 (β4); P28074 (β5); P20618 (β6), and P28070 (β7)) by using the Multiple_Alignment algorithm (Homology module, Accelrys, San Diego). Subsequently, the secondary structural prediction of human 20S proteasome was performed using the Structure Prediction and Sequence Analysis server PredictProtein (http://www.predictprotein.org/). The coordinates of the structurally conserved regions (α1: aa5-244; α2: aa3-234; α3: aa2-251; α4: aa3-240; α5: aa9-127 and aa134-241; α6: aa4-241; α7: aa6-245; β1: aa1-191; β2: aa1-220; β3: aa1-204; β4: aa1-199; β5: aa1-201; β6: aa1-213; β3: aa1-215) were accordingly assigned by the Structurally Conserved Regions (SCRs)-AssignCoords procedure (Homology Module, Insight 2005) using 5T0J as template structure. The lacking loop segment in the α5 subunit (aa128-133) was inserted by using the Generate Loops procedure. With the Generate Loops procedure, a peptide backbone chain is built between two conserved peptide segments using randomly generated values for all the loops' φ's and ψ's. The chain was defined starting from the N-terminal end of the loop being built; the Start and Stop Residues were defined as the SCR residues of the model protein at either end of the

loop itself. The geometry about the base was described by the four distances between Cα and N-termini of the Start residue and the Cα and C-termini of the Stop Residues. In the process of closing the loop, the values for the generated φ's and ψ's are adjusted until the four distance criteria are met. Specifically, a function was defined for the distances in terms of the dihedral angles (Scale Torsions: 60). The differences between the desired distances and their current values were minimized using a linearized Lagrange multiplier method. After a series of 1000 iterations, the loop was closed, except in the case where the distances between the ends of the loop were not respected (Convergence = 0.05). The geometry at the base of the loop is then checked for proper chirality. Finally, the loops were screened on the basis of steric overlap violations. All loops that are found to have unacceptable contacts were rejected. Since successive calculations can correct some bad contacts, a fairly large overlap factor was used (Internal and External overlap = 0.6). A bump check of the 10 generated loops together with the evaluation of their conformational energy were used as selection criteria. The lowest conformational energy loop presenting no steric overlap with the rest of the protein, was selected. Finally, the coordinates of the lacking N-terminal and C-terminal amino acids were assigned using the EndRepair command (Homology Module, Insight 2005). The obtained homology model was completed inserting the missing residue side chains by using the Replace command (Biopolymer module, Accelrys, San Diego). The obtained homology model was subjected to the same full energy minimization and structural check procedure previously described for the homology model of human 20S proteasome in the closed conformation, and, then, used for the subsequent dynamic docking studies. During the minimization, the whole disordered N- and C-terminals, the inserted loop region and the SCRs side chains were left free to move, whereas the SCRs backbone were fixed to avoid unrealistic results.

*Calculation of the chemical-physical properties of **2**.* The apparent pKa values were estimated by using the ACD/Percepta software.[7] The compound was considered in its cationic form in all calculations performed, as a consequence of the estimation of percentage of neutral/ionized forms computed at pH 7.4 (physiological value) and pH 7.2 (cytoplasmic value) using the Handerson−Hasselbalch equation. Atomic potentials were assigned using the CVFF force field, while the partial charges were assigned using the partial charges estimated by MNDO semi-empirical 1 SCF calculations.[8] The conformational space of compound was sampled through 200 cycles of simulated annealing (SA; $\varepsilon = 80*r$) followed by molecular mechanics (MM) energy minimization. During the SA procedure, the temperature is altered in time increments from an initial temperature to a final temperature by adjusting the kinetic energy of the structure (by rescaling the velocities of the atoms). The following protocol was applied: the system was heated to 1000 K over 2000 fs (time step of 1.0 fs); a temperature of 1000 K was applied to the system for 2000 fs (time step of 1.0 fs) to surmount torsional barriers; successively, temperature was linearly reduced to 300 K in 1000 fs with a decrement of 0.5 K/fs (time step of 1.0 fs). Resulting conformations were then subjected to MM energy minimization within Insight 2005 Discover 3 module (CVFF force field; $\varepsilon = 80*r$) until the maximum rms was less than 0.001 kcal/Å, using conjugate gradient[9] as the minimization algorithm. The resulting MM conformers were subsequently ranked by: i) conformational energy (ΔE from the global energy minimum < 5 kcal/mol), ii) interatomic distances between the charged nitrogen atoms, and iii) conformation of porphyrin ring. In order to properly analyze the electronic properties, the conformers, obtained from molecular dynamics and mechanics calculations, were subjected to a full geometry optimization through semiempirical calculations, using the quantum mechanical method PM7[10] in the Mopac2012 package [11]. The EF (Eigenvector Following routine) algorithm of geometry optimization was used,[12] with a GNORM value set to 0.01. To reach a full geometry optimization, the criterion for terminating all optimizations was increased by a factor of 100, using the keyword PRECISE. The resulting PM7 conformers were subsequently ranked as reported above for MM conformers.

Docking studies on human 20S proteasome in complex with **2**. According to the bioinformatic analysis performed on 20S proteasome and the conformational analysis of **2**, we selected the α4-α5 groove as starting point for the docking studies of **2**. The putative starting complex was subjected to dynamic docking studies (Affinity, SA_Docking; Insight2005, Accelrys, San Diego). In particular, a docking methodology, which considers all the systems flexible (i.e., ligand and protein), was used. Flexible docking was achieved using the Affinity module in the Insight 2005 suite, setting the SA_Docking procedure[13] and using the Cell Multipole method for nonbond interactions.[14]

The binding domain area was defined as a subset including all residues of human 20S proteasome. All atoms included in the binding domain area were left free to move during the entire course of docking calculations, whereas, in order to avoid unrealistic results, a tethering restraint was applied on the SCRs of protein.

To identify SCRs, the human 20S proteasome sequences were analyzed using the Structure Prediction and Sequence Analysis server PredictProtein (http://www.predictprotein. org/). In α1 subunits, 6 α-helix and 10 β-sheet secondary structures were predicted to be highly conserved (α1, aa23−33; α2, aa85−104; α3, aa111−128; α4, aa172−183; α5, aa191−207; α6, aa232−243; β1, aa13−16; β2, aa38−43; β3, aa48−53; β4, aa68−72; β5, aa76−80; β6, aa135-143; β7, aa150-154; β8, aa160-168; β9, aa215-222; β10, aa226-229). In α2 subunits, 6 α-helix and 10 β-sheet secondary structures were predicted to be highly conserved (α1, aa20−30; α2, aa81−100; α3, aa107−124; α4, aa167−178; α5, aa184−198; α6, aa223−231; β1, aa9−13; β2, aa34−39; β3, aa44−49; β4, aa66−68; β5, aa72−76; β6, aa131-139; β7, aa145-149; β8, aa155-163; β9, aa208-214; β10, aa219-220). In α3 subunits, 6 α-helix and 10 β-sheet secondary structures were predicted to be highly conserved (α1, aa18−29; α2, aa80−100; α3, aa107−124; α4, aa168−178; α5, aa186−200; α6, aa230−248; β1, aa10−12; β2, aa33−38; β3, aa43−48; β4, aa66−68; β5, aa72−76; β6, aa131-139; β7, aa146-150; β8, aa157-164; β9, aa211-217; β10, aa224-227). In α4 subunits, 6 α-helix and 10 β-sheet secondary structures were predicted to be highly conserved (α1, aa17−27; α2, aa78−97; α3, aa104−121; α4, aa165−176; α5, aa183−198; α6, aa222−243; β1, aa6−10; β2, aa31−36; β3, aa41−46; β4, aa62−65; β5, aa69−73; β6, aa128-136; β7, aa143-147; β8, aa154-161; β9, aa206-212; β10, aa217-219). In α5 subunits, 6 α-helix and 10 β-sheet secondary structures were predicted to be highly conserved (α1, aa22−32; α2, aa83−102; α3, aa109−120; α4, aa174−185; α5, aa191−206; α6, aa231−240; β1, aa11−15; β2, aa36−41; β3, aa46−51; β4, aa66−70; β5, aa74−78; β6, aa138-146; β7, aa152-156; β8, aa162-170; β9, aa215-221; β10, aa226-228). In α6 subunits, 6 α-helix and 10 β-sheet secondary structures were predicted to be highly conserved (α1, aa19−30; α2, aa79−98; α3, aa105−122; α4, aa165−176; α5, aa184−199; α6, aa226−236; β1, aa10−13; β2, aa35−39; β3, aa45−49; β4, aa62−66; β5, aa70−74; β6, aa129-137; β7, aa143-147; β8, aa154-161; β9, aa210-216; β10, aa221-223). In α7 subunits, 6 α-helix and 10 β-sheet secondary structures were predicted to be highly conserved (α1, aa22−32; α2, aa83−102; α3, aa109−124; α4, aa170−180; α5, aa187−202; α6, aa229−245; β1, aa13−15; β2, aa36−41; β3, aa46−51; β4, aa67−70; β5, aa74−78; β6, aa133-141; β7, aa148-152; β8, aa160-166; β9, aa212-219; β10, aa224-227). In β1 subunits, 5 α-helix and 11 β-sheet secondary structures were predicted to be highly conserved (α1, aa52−69; α2, aa76−90; α3, aa132-143; α4, aa149−166; α5, aa191−200; β1, aa2−8; β2, aa12−16; β3, aa25−27; β4, aa34−37; β5, aa41−45; β6, aa95-103; β7, aa110-114; β8, aa120-122; β9, aa124-128; β10, aa174-180; β11, aa185-189). In β2 subunits, 5 α-helix and 12 β-sheet secondary structures were predicted to be highly conserved (α1, aa52−67; α2, aa76−90; α3, aa131-142; α4, aa148−165; α5, aa194−201; β1, aa2−8; β2, aa12−17; β3, aa25−27; β4, aa33−37; β5, aa41−45; β6, aa95-103; β7, aa109-113; β8, aa123-127; β9, aa173-179; β10, aa184-186; β11, aa210-213; β12, aa215-225). In β3 subunits, 4 α-helix and 10 β-sheet secondary structures were predicted to be highly conserved (α1, aa56−76; α2, aa83−97; α3, aa142-152; α4, aa159−175; β1, aa9−14; β2, aa18−24; β3, aa32−34; β4, aa41−44; β5, aa48−52; β6, aa103-111; β7, aa119-123; β8, aa134-138; β9, aa184-190; β10, aa194-199). In β4 subunits, 5 α-helix and

10 β-sheet secondary structures were predicted to be highly conserved (α1, aa32−35; α2, aa50−70; α3, aa77−93; α4, aa136-147; α5, aa153−170; β1, aa3−8; β2, aa12−17; β3, aa26−27; β4, aa36−38; β5, aa42−46; β6, aa99-107; β7, aa114-118; β8, aa129-132; β9, aa178-184; β10, aa189-190). In β5 subunits, 6 α-helix and 11 β-sheet secondary structures were predicted to be highly conserved (α1, aa31−34; α2, aa49−69; α3, aa76−91; α4, aa132-143; α5, aa149−166; α6, aa189−200; β1, aa2−8; β2, aa12−16; β3, aa25−26; β4, aa35−37; β5, aa41−45; β6, aa96-104; β7, aa110-114; β8, aa120-122; β9, aa124-128; β10, aa174-180; β11, aa185-186). In β6 subunits, 4 α-helix and 11 β-sheet secondary structures were predicted to be highly conserved (α1, aa58−77; α2, aa85−99; α3, aa142-152; α4, aa168−184; β1, aa5−6; β2, aa11−16; β3, aa20−26; β4, aa34−36; β5, aa42−46; β6, aa50-54; β7, aa105-113; β8, aa120-124; β9, aa134-138; β10, aa193-199; β11, aa203-210). In β7 subunits, 6 α-helix and 10 β-sheet secondary structures were predicted to be highly conserved (α1, aa39−41; α2, aa57−77; α3, aa85-100; α4, aa143−154; α5, aa162−179; α6, aa207−216; β1, aa10−16; β2, aa20−25; β3, aa33−35; β4, aa42−45; β5, aa49−53; β6, aa107-115; β7, aa121-125; β8, aa136-139; β9, aa187-193; β10, aa198-199). Accordingly, for the alpha-helices, the distance between backbone hydrogen bond donors and acceptors was restrained within 2.5 Å. On the other hand, for the beta-sheets, the φ and ψ torsional angles, according to the parallel or anti-parallel conformation, were restrained within -119° and +113°, or -139° and +135°, respectively (Restrain command; Discover_3 module, Accelrys, San Diego). According to the reliability index values obtained from secondary structure prediction analysis, the following set of restraint force constants was used: i) force constants of 1 kcal/mol/$Å^2$-10 kcal/mol/$Å^2$ for reliability index values from 0 to 3, ii) force constants of 10 kcal/mol/$Å^2$-100 kcal/mol/$Å^2$ for reliability index values from 4 to 6, and iii) force constants of 100 kcal/mol/$Å^2$-1000 kcal/mol/$Å^2$ for reliability index values from 7 to 9.

The docking protocol included a Monte Carlo based conformational search of the ligand within the obtained homology models of human 20S proteasome (i.e., closed and open conformation) for the random generation of a maximum of 20 acceptable complexes. During the first step, starting from the previously obtained roughly docked structures, the ligand was moved by a random combination of translation, rotation, and torsional changes to sample both the conformational space of the ligand and its orientation with respect to the protein (MxRChange = 3 Å; MxAngChange = 180°). During this step, van der Waals (vdW) and Coulombic terms were scaled to a factor of 0.1 to avoid very severe divergences in the vdW and Coulombic energies. If the energy of a complex structure resulting from random moves of the ligand was higher by the energy tolerance parameter than the energy of the last accepted structure, it was not accepted for minimization. To ensure a wide variance of the input structures to be successively minimized, an energy tolerance value of 10^6 kcal/mol from the previous structure was used. After the energy minimization step (conjugate gradient; 2500 iterations; ε = 1), the energy test, with an energy range of 50 kcal/mol, and a structure similarity check (rms tolerance = 0.3 kcal/Å) was applied to select the 20 acceptable structures. Each subsequent structure was generated from the last accepted structure. Following this procedure, the resulting docked structures were ranked by their conformational energy and were analyzed considering the nonbond interaction energies between the ligand and the enzyme (vdW and electrostatic energy contribution; Group Based method; CUT_OFF = 10; ε = 2*r; Discover_3 Module of Insight2005). Finally, in order to test the thermodynamic stability of the resulting docked complexes, these latter were subjected also to a molecular dynamics SA protocol using the Cell_Multipole method for nonbond interactions and the dielectric constant of the water (ε = 80*r). A tethering restraint was applied on the SCRs of the complex. The set of structural restraints applied was the same as for previous docking calculations. The protocol included 5 ps of a dynamic run divided in 50 stages (100 fs each) during which the temperature of the system was linearly decreased from 500 to 300 K (Verlet velocity integrator; time step = 1.0 fs). In simulated annealing, the temperature is altered in time increments from an initial temperature to a final temperature. The temperature is changed by adjusting the kinetic energy of the structure (by rescaling the velocities

of the atoms). Molecular dynamics calculations were performed using a constant temperature and constant volume (NVT) statistical ensemble, and the direct velocity scaling as temperature control method (temp window = 10 K). In the first stage, initial velocities were randomly generated from the Boltzmann distribution, according to the desired temperature, while during the subsequent stages initial velocities were generated from dynamics restart data. The temperature of 500 K was applied with the aim of surmounting torsional barriers, thus allowing an unconstrained rearrangement of the "ligand" and the "protein" binding site (initial vdW and Coulombic scale factors = 0.1). Successively temperature was linearly reduced to 300 K in 5 ps, and, concurrently, the vdW and Coulombic scale factors have been similarly increased from their initial values (0.1) to their final values (1.0). A final round of 10^5 minimization steps ($\varepsilon = 80*r$) followed the last dynamics steps, and the minimized structures were saved in a trajectory file. In order to allow the whole relaxation of the protein, the resulting annealed complexes were then subjected to MM energy minimization without restraints (Steepest Descent algorithm; $\varepsilon = 80*r$) until the maximum RMS derivative was less than 0.5 kcal/Å (Module Discover; Insight 2005). The ligand/enzyme complexes thus obtained were ranked by their conformational energy and analyzed considering the nonbond interaction energies between the ligand and the enzyme (vdW and electrostatic energy contribution; Group Based method; CUT_OFF = 10; $\varepsilon = 2*r$; Discover_3 Module of Insight2005). The complex with the best compromise between the non-bond interaction energies obtained by Monte Carlo and SA calculations was selected as the structure representing the most probable binding mode.

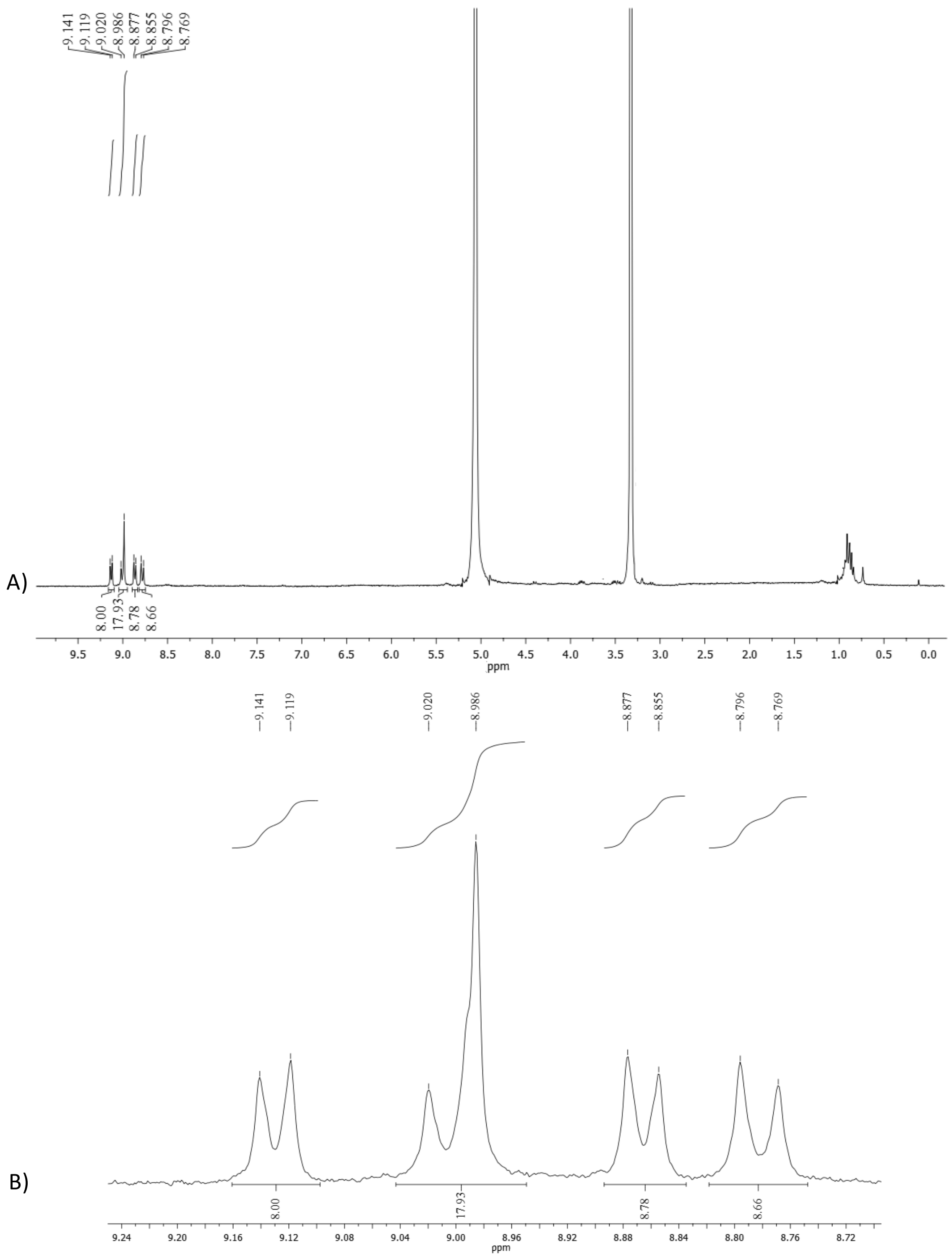

Figure S1. A) ^{1}H-NMR spectrum (MeOD+TFA) of porphyrin **2***. In B) the expansion of the aromatic region is reported.

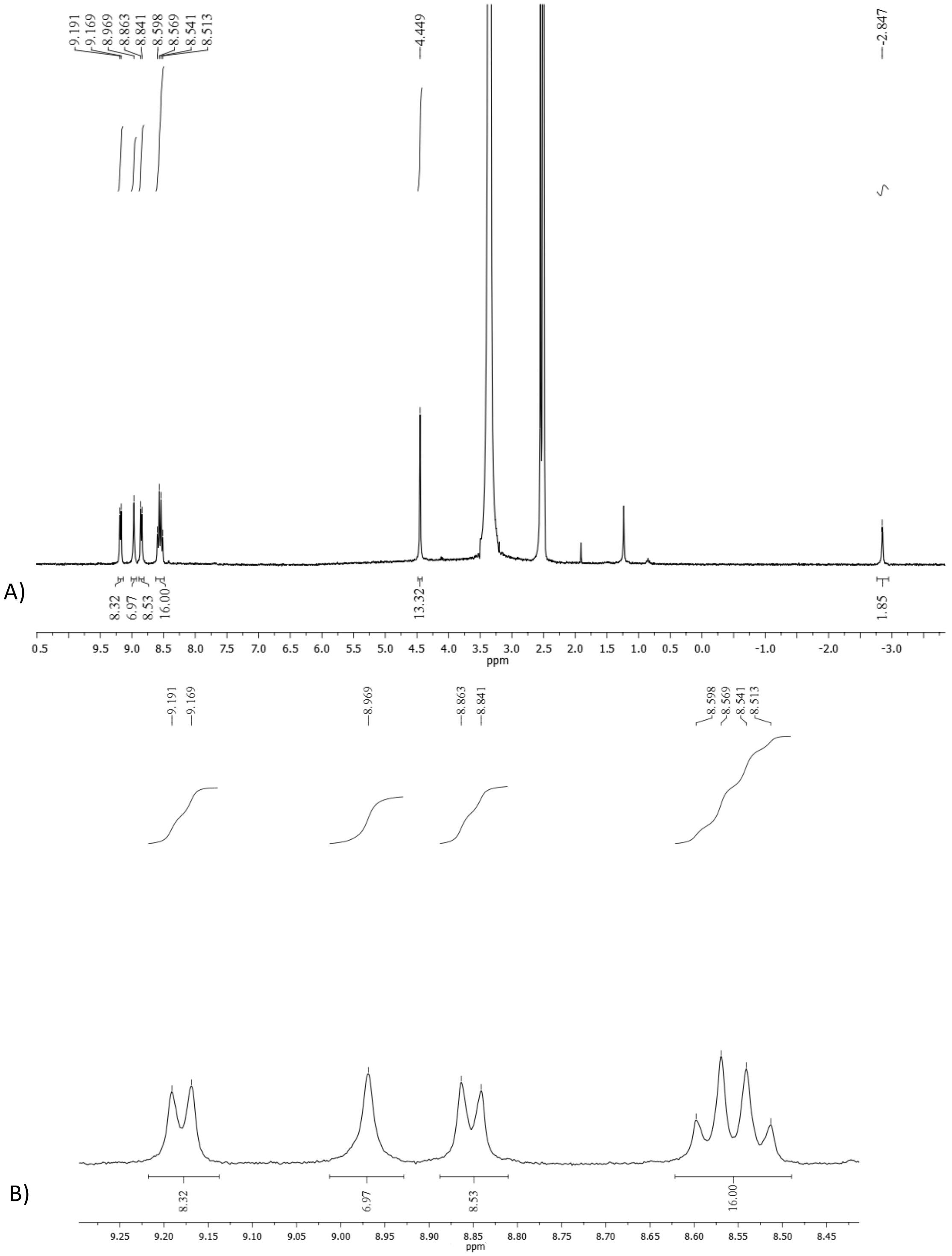

Figure S2. A) ^{1}H-NMR spectrum (DMSO-d_6) of porphyrin **2**. In B) the expansion of the aromatic region is reported.

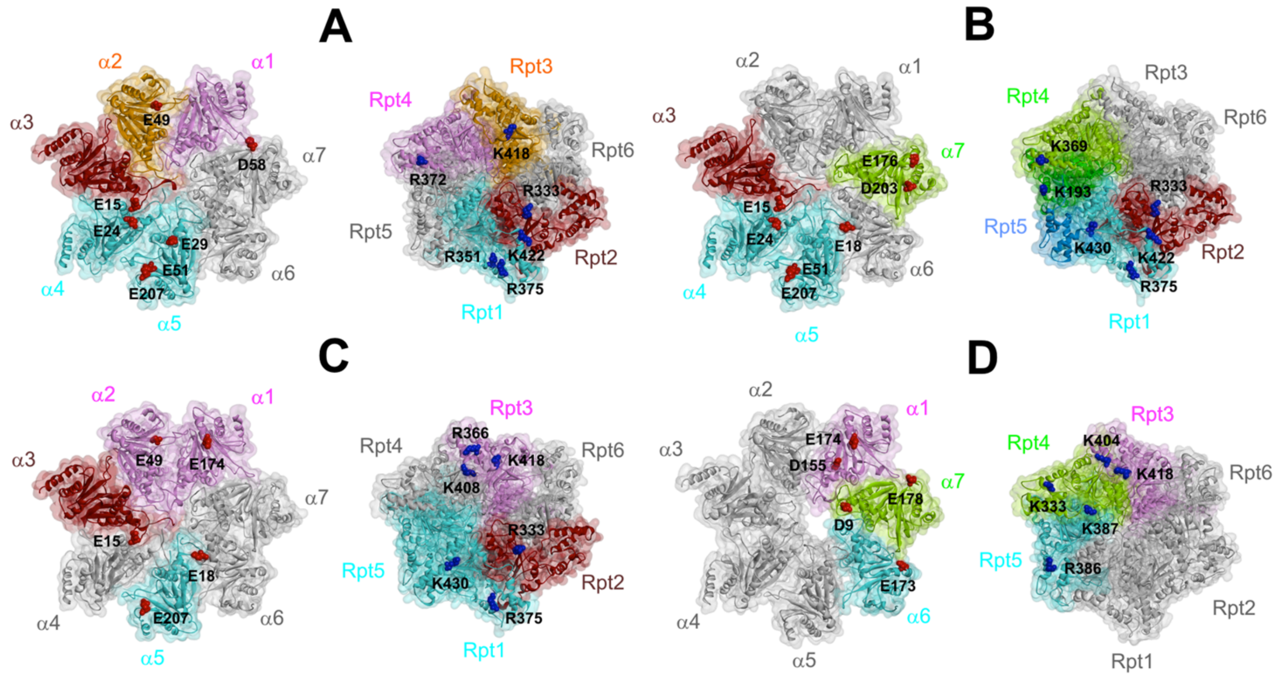

Figure S3. Ionic interactions occurring between human 20S proteasome and 19S RP complex: top view of the α1-α7 ring of human 20S (left) is reported next to the bottom view of the Rpt1-6 subunits of 19S (right). (A) S_A (PDB ID: 5T0G), (B) S_B (PDB ID: 5T0H), (C) S_C (PDB ID: 5T0I), and (D) S_D (PDB ID: 5T0J) conformational/functional states. Proteins are displayed as solid ribbons and their van der Waals volume is displayed as transparent surface. The residues involved in ionic interactions between 20S (negative: red) and 19S (positive: blue) are displayed as CPK and labeled. The α subunits of 20S containing negatively charged interacting residue(s) are colored by the same color as the Rpt subunits of 19S containing the corresponding positively charged interacting residue(s). The rest of the 20S and 19S subunits are colored in gray.

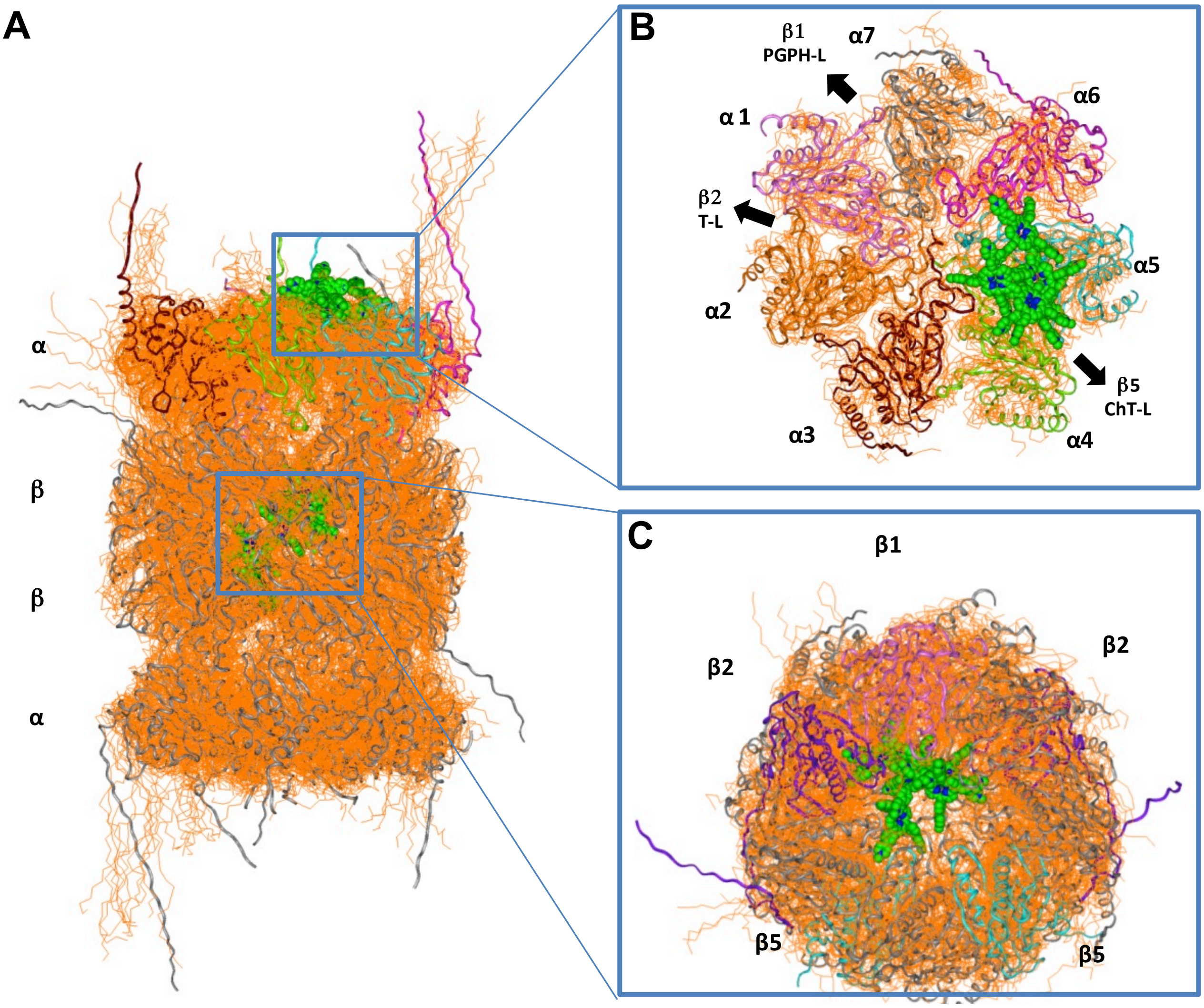

Figure S4. A) Dynamic docking results obtained for **2** using as starting structure the closed conformation of human 20S proteasome. B) Top view of dynamic docking results without the two rings of β subunits and the second ring of α subunits. C) Top view of dynamic docking results without the first ring of α subunits. The backbone of the starting complex is displayed as solid ribbons and colored in pink (α1), orange (α2), brown (α3), light green (α4), cyan (α5), magenta (α6), and gray (α7, α subunits of the second ring and all β subunits). The backbone of the calculated complexes is displayed as line ribbons and colored in orange. The porphyrin ligands are colored by atom type (C: green and N: blue) and displayed as CPK. In B the α subunits and the catalytic β subunits are labeled. In C the catalytic β subunits are labeled and colored in pink (β1), violet (β2), and cyan (β5). The last six generated complexes (each docked complex is generated from the last accepted structure, see the Experimental Section for details) presented the ligand positioned in the substrate channel at the level of the first β-ring, or at the interface between the first and the second β-ring (evidenced in C); however, the binding to the α4-α5 groove (evidenced in B) is favored according to the calculated non-bond interactions energies.

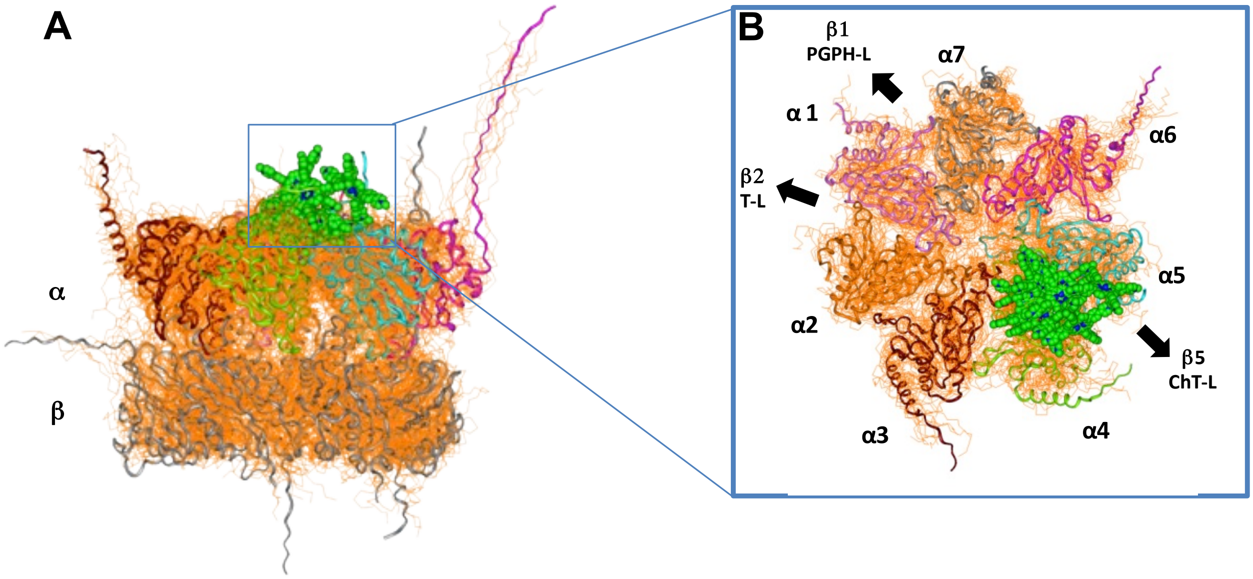

Figure S5. A) Dynamic docking results obtained for **2** using as starting structure the open conformation of human 20S proteasome. B) Top view of dynamic docking results without the ring of β subunits. The backbone of the starting complex is displayed as solid ribbons and colored in pink (α1), orange (α2), brown (α3), light green (α4), cyan (α5), magenta (α6), and gray (α7 and β subunits). The backbone of the calculated complexes is displayed as line ribbons and colored in orange. The porphyrin ligands are colored by atom type (C: green and N: blue) and displayed as CPK. In B the α subunits and the catalytic β subunits are labeled.

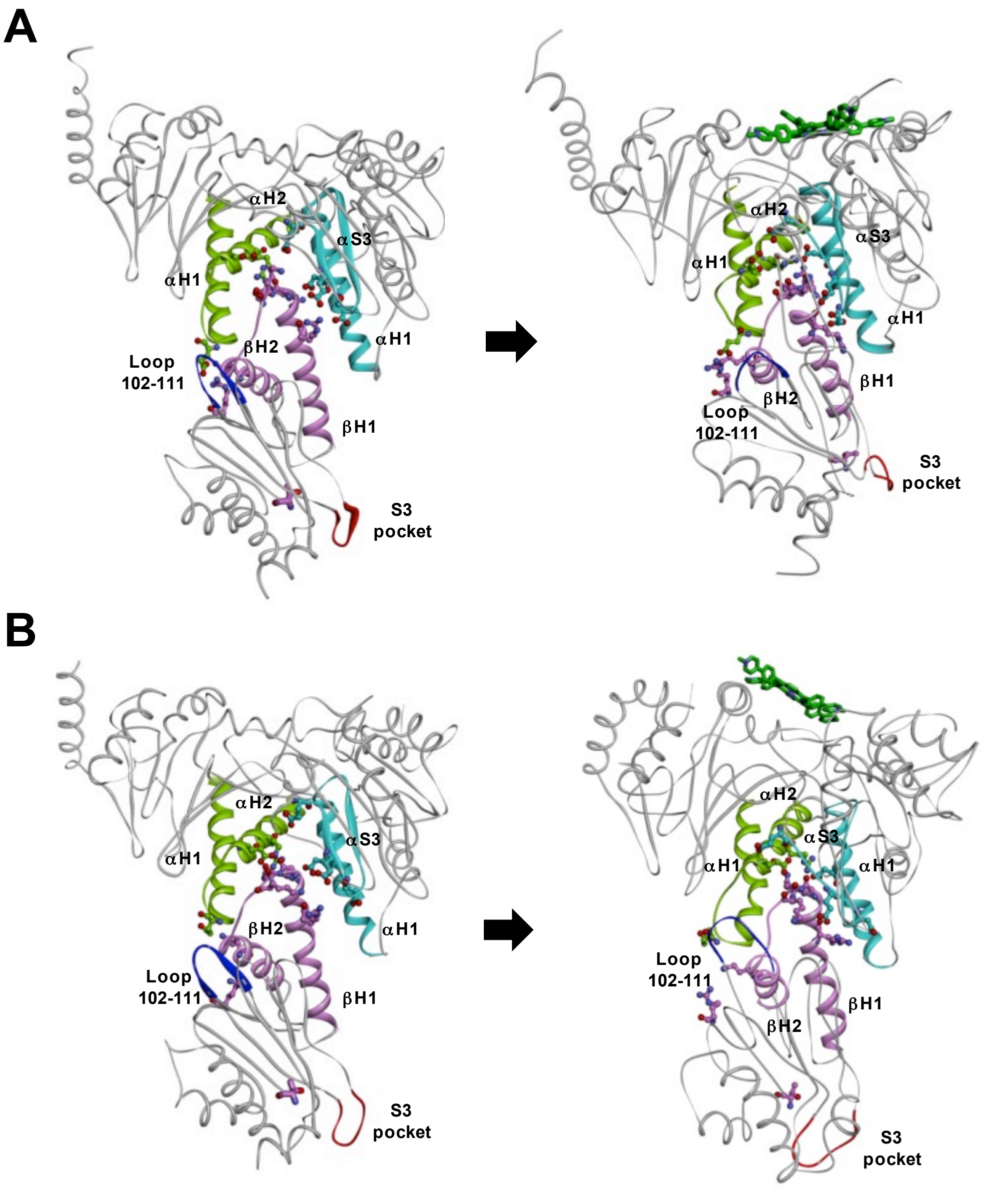

Figure S6. α4, α5, and β5 subunits before (left) and after (right) docking calculations in complex with **2**. (A) Starting from the 20S closed state. (B) Starting from the 20S open state. The protein is displayed as gray ribbons. αH1 and αH2 (α4 subunit; light green), αS3, αS4 and αH1 (α5 subunit; cyan), βH1 and βH2 (β5 subunit; pink), the loop region aa102-111 (β5 subunit; blue), and the S3 pocket (β5 subunit; red), are evidenced as solid ribbons. Key residues are displayed in ball&sticks. **2** is displayed as stick and colored by atom type (C = green and N = blue). In (A) the conformational changes at α4-α5 groove caused, in the region at the interface among the subunits α4, α5 and β5, the break of the ionic interaction between E65 (αS3 strand of subunit α5) and K71 (loop between the helices βH1 and βH2 of subunit β5) which moved to interact with E69 (αS3 strand of subunit α5). In turn, this change moved the helix αH2 (aa104-121) of the subunit α4 toward both the subunit β5, determining the formation of a hydrogen bond between R109 (α4) and N70 (β5), and the subunit α5 determining the formation of an ionic interaction between R109 (α4) and D90 (α5). Moreover, this movement modified the positioning of the βH2 helix with respect the loop region (aa102-111) within the subunit β5.

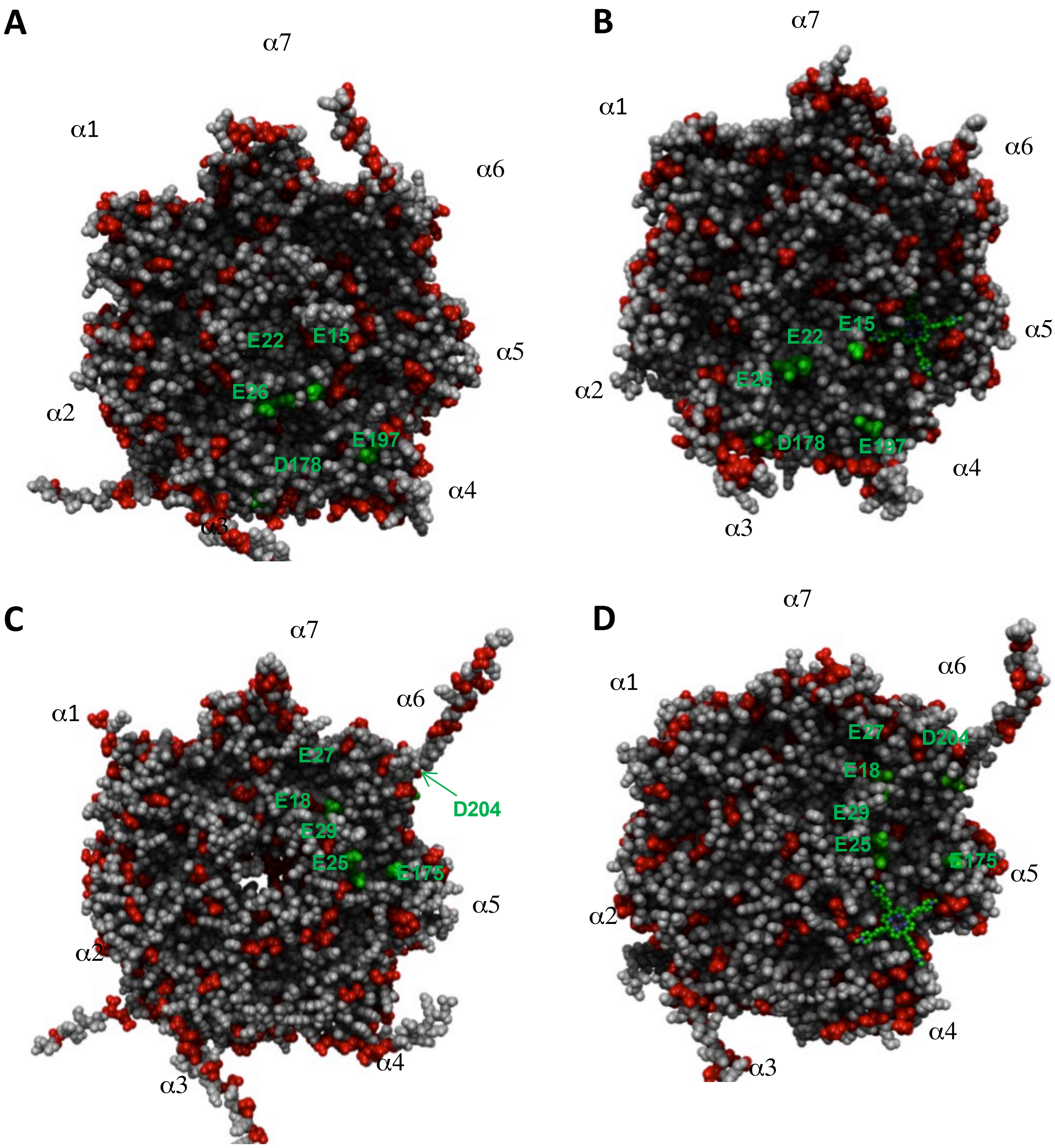

Figure S7. Top view of: A) human 20S closed state, B) human 20S in complex with **2** (Cplx_3), C) human 20S open state, and D) human 20S in complex with **2** (Cplx_9). In B and D **2** is displayed in ball&stick and colored by atom type (C = green; N = blue). The human 20S proteasome is displayed as CPK and colored in gray. All negatively charged residues are colored in red with the exception of those present in the groove α3-α4 (A and B) or in the groove α5-α6 (C and D) which are evidenced in green and labelled. Hydrogens are omitted for sake of clarity.

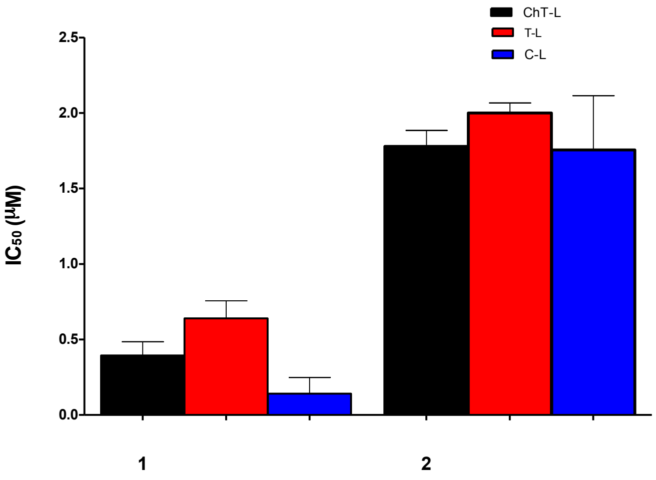

IC_{50}	1	2
ChT-L	0,393±0,09	1,77±0,07
T-L	0,64 ±0,11	2,00±0,06
PGPH-L	0,140 ±0,10	1,75±0,36

Figure S8. Comparison of the IC_{50} values of **1** and **2** determined for the ChT-L (black), T-L (red) and PGPH-L (blue) peptidase activities of the CP.

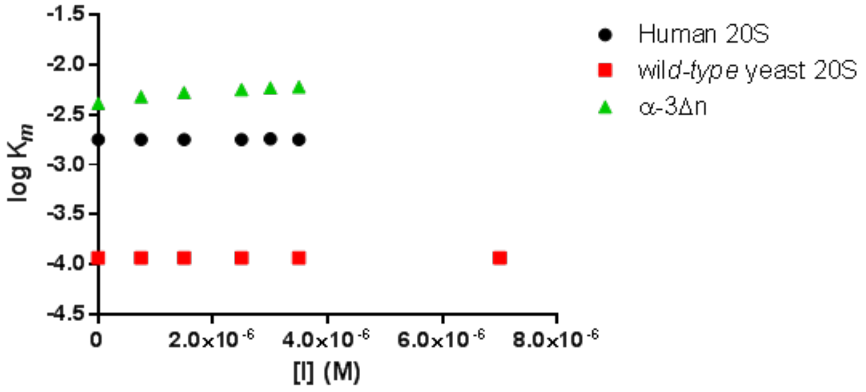

Figure S9A. Porphyrin concentration dependence of K_m, as from fitting of data, reported in Figs. 4A-C, according to Eqs. (S1) and (S3).

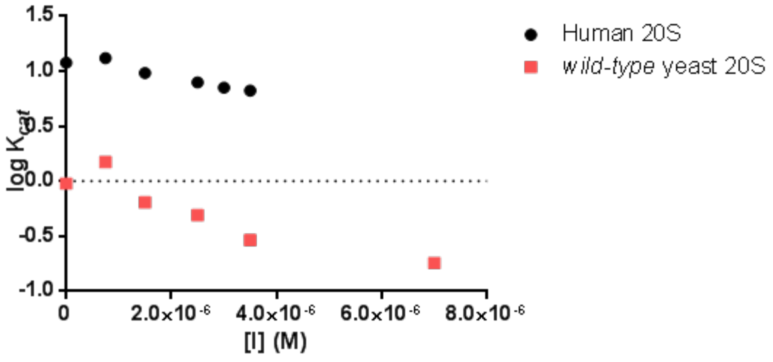

Figure S9B. Porphyrin concentration dependence of k_{cat}, as from fitting of data, reported in Figs. 4A-C, according to Eqs. (S1) and (S3).

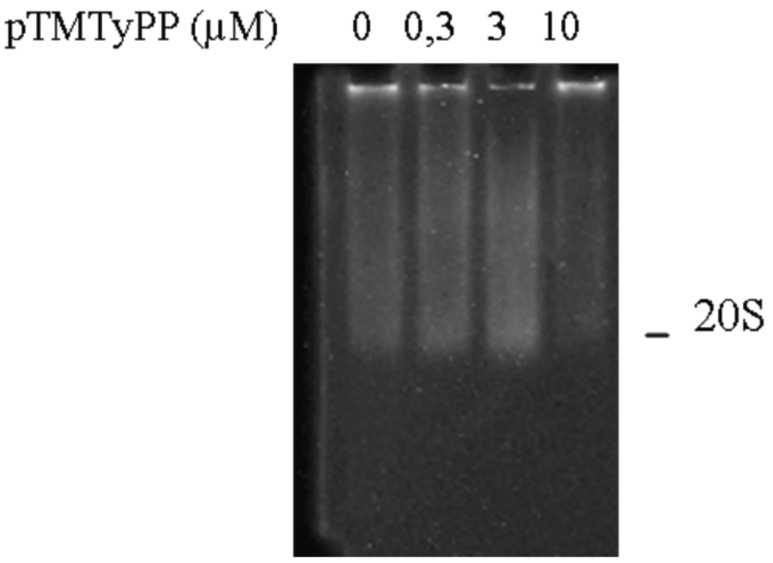

Figure S10. The purified human 20S proteasome (1μg) was incubated with various concentrations (namely 0, 0.3 μM, 3 μM and 10 μM, from left to right) of pTMPyPP4 and separated by native gel electrophoresis. The particles were probed with 100 μM Suc-LLVY-AMC.

Table S1. Ionic interactions between human 20S (negative) and PA28α (positive) obtained using as reference structure the X-ray complex of yeast 20S proteasome with Trypanosoma PA26 (PDB ID: 1ZTQ).

yeast 20S proteasome (negative)	*Trypanosoma Brucei PA26*[a] *(positive)*	*human 20S proteasome (negative)*	*human PA28α (positive)*[a]
E22 (α1)	K100 (Subunit 7)	E19 (α1)	R141 (Subunit 7)
-	-	E26 (α1)	K245 (Subunit 7)
-	-	E23 (α2)	K245 (Subunit 1)
E16 (α3)	K100 (Subunit 2)	E15 (α3)	R141 (Subunit 2)
-	-	E22 (α3)	K245 (Subunit 2)
D14 (α4)	K100 (Subunit 3)	D13 (α4)	R141 (Subunit 3)
-	-	E20 (α4)	K245 (Subunit 3)
E18 (α5)	K100 (Subunit 4)	E18 (α5)	R141 (Subunit 4)
-	-	E25 (α5)	K245 (Subunit 4)
-	-	E23 (α6)	K245 (Subunit 5)
D17 (α7)	K100 (Subunit 6)	D18 (α7)	R141 (Subunit 6)
-	-	E25 (α7)	K245 (Subunit 6)

[a] PA26 and PA28 subunits are numbered according to PDB structures 1ZTQ and 1AVO, respectively.

Table S2. Ionic interactions between human 20S (negative) and PA200 (positive) obtained using the X-ray complex of yeast 20S proteasome with yeast Blm10 (PDB ID: 4V7O) as reference structure.

yeast 20S proteasome (negative)	***yeast Blm10 (positive)***	***human 20S proteasome (negative)***	***human PA200 (positive)***
-	-	E175 (α2)	K1025
-	-	E200 (α2)	K711<br>K878
-	-	E58 (α3)	K1069
-	-	D202 (α3)	R1074
-	-	E209 (α3)	K1069
E173(α4)	R1798	E170(α4)	R1502
E7 (α5)	K589	E7 (α5)	K411
D9 (α5)	K2112	D9 (α5)	R1811
E18 (α5)	K2111	E18 (α5)	R1810
E25 (α5)	K2112	E25 (α5)	R1811
E27 (α6)	K2111	E27 (α6)	R1810
D203 (α6)	R2048	E202 (α6)	R1742
-	-	D204 (α6)	R1740

Table S3. Ionic interactions between human 20S (negative) and the RP protein complex 19S (positive) in the S_{A-D} states (PDB IDs: 5T0G; 5T0H; 5T0I; 5T0J).

human 20S proteasome (negative)	***human 19S (positive)***
D58 (α1)	R372 (Rpt4)
D155 (α1)	K418 (Rpt3)
	R366 (Rpt3)
E174 (α1)	K408 (Rpt3)
	K404 (Rpt3)
D236 (α1)	K36 (Rpn5)
E244 (α1)	K36 (Rpn5)
E49 (α2)	K418 (Rpt3)
E183 (α2)	K121 (Rpn6)
E185 (α2)	R122 (Rpn6)
D228 (α2)	K84 (Rpn6)
E15 (α3)	R333 (Rpt2)
E24 (α4)	K422 (Rpt1)
E18 (α5)	K430 (Rpt5)
E29 (α5)	R351 (Rpt1)
E51 (α5)	R375 (Rpt1)
E207 (α5)	R375 (Rpt1)
E173 (α6)	R386 (Rpt5)
D9 (α7)	K387 (Rpt4)
E176 (α7)	K369 (Rpt4)
D203 (α7)	K193 (Rpt5)

Table S4. Identified clusters of four planar negatively charged amino acids present on the α-ring surface of human 20S proteasome in closed (PDB ID: 4R3O) and open conformation (PDB ID: 5T0J) and related inter-residue distances (Cα).

	**PDB ID 4R3O**				**PDB ID 5T0J**			
**Groove**	**d1 (Å)**	**d2(Å)**	**d3(Å)**	**d4(Å)**	**d1 (Å)**	**d2(Å)**	**d3(Å)**	**d4(Å)**
**α1-α2**	20.45<br>D18(α7)-E174(α1)	24.17<br>E174(α1)-E200(α2)	22.17<br>E200(α2)-E19(α1)	20.19<br>E19(α1)-D18(α7)	22.02<br>D18(α7)-E174(α1)	26.23<br>E174(α1)-E200(α2)	23.02<br>E200(α2)-E19(α1)	20.11<br>E19(α1)-D18(α7)
**α4-α5 (I)**	24.45<br>E15(α3)-E170(α4)	20.63<br>E170(α4)-E207(α5)	22.46<br>E207(α5)-D13(α4)	19.09<br>D13(α4)-E15(α3)	26.48<br>E15(α3)-E170(α4)	22.04<br>E170(α4)-E207(α5)	24.75<br>E207(α5)-D13(α4)	19.28<br>D13(α4)-E15(α3)
**α4-α5 (II)**	24.45<br>E15(α3)-E170(α4)	20.63<br>E170(α4)-E207(α5)	20.67<br>E207(α5)-E29(α5)	24.03<br>E29(α5)-E15(α3)	26.48<br>E15(α3)-E170(α4)	22.04<br>E170(α4)-E207(α5)	21.97<br>E207(α5)-E29(α5)	25.03<br>E29(α5)-E15(α3)
**α4-α5 (III)**	19.54<br>E24(α4)-E170(α4)	20.63<br>E170(α4)-E207(α5)	22.46<br>E207(α5)-D13(α4)	18.63<br>D13(α4)-E24(α4)	21.28<br>E24(α4)-E170(α4)	22.04<br>E170(α4)-E207(α5)	24.75<br>E207(α5)-D13(α4)	18.56<br>D13(α4)-E24(α4)
**α4-α5 (IV)**	19.54<br>E24(α4)-E170(α4)	20.63<br>E170(α4)-E207(α5)	20.67<br>E207(α5)-E29(α5)	22.41<br>E29(α5)-E24(α4)	21.28<br>E24(α4)-E170(α4)	22.04<br>E170(α4)-E207(α5)	21.97<br>E207(α5)-E29(α5)	23.98<br>E29(α5)-E24(α4)

Table S5. Non-bond interaction energies (kcal/mol) of the 20S-**2** complexes obtained by Monte Carlo and SA calculations using as starting structure the closed conformation of 20S.

**Cplx**	**Non-bond interaction energies (kcal/mol)**	
	Monte Carlo Simulation	Simulated Annealing Simulation
**1**	-42.363	-66.518
**2**	-42.948	-81.754
**3**[a]	-53.260	-76.404
**4**	-50.670	-50.246
**5**	-25.029	-60.942
**6**	-6.877	-57.008
**7**	-6.649	-65.689
**8**	-4.466	-21.349
**9**	-6.976	-63.463
**10**	-17.344	-59.432
**11**	-3.415	-50.117

[a]Selected complex

Table S6. Non-bond interaction energies (kcal/mol) of the 20S-**2** complexes obtained by Monte Carlo and SA calculations using as starting structure the open conformation of 20S.

**Cplx**	**Non-bond interaction energies (kcal/mol)**	
	Monte Carlo Simulation	Simulated Annealing Simulation
**1**	-14.601	-56.056
**2**	-6.522	-55.024
**3**	-5.914	-0.147
**4**	-0.689	-62.805
**5**	0.672	-16.006
**6**	0.075	-42.347
**7**	-0.904	-70.354
**8**	-0.234	-60.497
**9**[a]	-3.970	-66.822
**10**	-1.321	-61.119
**11**	-1.646	-13.116

[a]Selected complex

Table S7. Summary of Molprobity results obtained for the experimentally determined structures of human 20S proteasome (PDB IDs: 4R3O and 5T0J), the homology models, the calculated porphyrin/20S human proteasome complexes.

Structure	Residues favored regions	Residues allowed regions	Residues outliers	Poor rotamers
X-ray (4R3O)	94.9%	4.3%	0.8%	4.3%
Cryo-EM (5T0J)	92.1%	7.7 %	0.2%	0.9%
Homology model (Closed Conformation)	94.8%	4.3%	0.9%	4.1%
Homology model (Open Conformation)	91.4%	8.2%	0.4%	1.3%
Cplx_3 (Annealed complex starting from the closed conformation)	78.9%	17.6%	3.5%	2.1%
Cplx_9 (Annealed complex starting from the open conformation)	77.4%	19.5%	3.1%	1.8%

Table S8. Ligand-residue non-bond interaction energies (kcal/mol) of the **2**-20S complex (Cplx_3) obtained by docking calculations using as starting structure the closed conformation of 20S.

20S amino acids	Subunit	Non-bond interaction Energy (kcal/mol)
S13	α3	-0.116
P14	α3	-1.060
E15[a]	α3	-0.312
G16	α3	-0.032
F10	α4	-0.197
D13[a]	α4	-1.729
G14	α4	-1.943
H15	α4	-5.439
L16	α4	-3.767
F17	α4	-1.748
V19	α4	-0.163
E20[a]	α4	-4.234
Y21	α4	-1.432
E24[a]	α4	-3.618
K166	α4	0.916
S167	α4	-0.128
E170[a]	α4	-4.907
K174	α4	1.688
M1	α5	0.823
R20	α5	0.949
E25[a]	α5	-3.610
Y26	α5	-4.008
E29[a]	α5	-5.828
A30	α5	-3.191
I31	α5	-0.343
L33	α5	-2.789
G34	α5	-3.326
S35	α5	-0.706
T36	α5	-0.282
R53	α5	-2.132
I54	α5	-1.922
T55	α5	-4.399
S56	α5	-0.367
P57	α5	-0.308
I64	α5	-0.066
G80	α5	-0.058
L81	α5	-1.589
G171	α5	-0.154
S172	α5	-2.549
A173	α5	-0.552
S174	α5	-0.291
L181	α5	-0.398
Q204	α5	-0.328
V205	α5	-2.274
M206	α5	-1.926
E207[a]	α5	-5.360
E208	α5	-0.823
K209	α5	1.094

[a] Negatively charged residues involved in ionic interaction with RPs.

Table S9. Inter-residues distances (Cα) of the negatively charged residues present in the α4-α5 groove of 20S in closed and open conformation as well as of 20S in complex with **2**.

**Residues**	**Distance (Å)**			
	20S closed conformation	20S open conformation	**2**-20S Cplx_3	**2**-20S Cplx_9
E15(α3)-E170(α4)	24.45	26.48	19.54	22.96
E170(α4)-E207(α5)	20.63	22.04	16.36	22.07
E207(α5)-D13(α4)	22.46	24.75	21.10	23.17
D13(α4)-E15(α3)	19.09	19.28	16.97	15.89
E207(α5)-E29(α5)	20.67	21.97	18.90	23.34
E29(α5)-E15(α3)	24.03	25.03	24.22	22.35
E24(α4)-E170(α4)	19.54	21.28	17.35	15.24
D13(α4)-E24(α4)	18.63	18.56	19.69	18.10
E29(α5)-E24(α4)	22.41	23.98	24.04	24.16

Table S10. Inter-residues distances (Cα) of the negatively charged residues present in the putative binding site for an additional ligand molecule at the α3-α4 groove of the docked structure of human 20S in complex with **2** (Cplx_3).

Site	Distances (Å)			
	d1	d2	d3	d4
α face groove α3-α4 (I)	19.83 E26(α3)-D178(α3)	27.60 D178(α3)-E197(α4)	21.35 E197(α4)-E15(α3)	21.62 E15(α3)-E26(α3)
α face groove α3-α4 (II)	23.70 E22(α3)-D178(α3)	27.60 D178(α3)-E197(α4)	21.35 E197(α4)-E15(α3)	16.83 E15(α3)-E22(α3)

Table S11. Ligand-residue non-bond interaction energies (kcal/mol) of the **2**-20S complex (Cplx_9) obtained by docking calculations using as starting structure the open conformation of 20S.

20S amino acids	Subunit	Non-bond interaction Energy (kcal/mol)
P12	α4	-1.048
D13[a]	α4	-2.680
G14	α4	-1.420
H15	α4	-4.893
L16	α4	-1.738
F17	α4	-3.192
V19	α4	-0.244
E20[a]	α4	-6.590
Y21	α4	-4.413
E24[a]	α4	-5.961
G162	α4	-0.023
R163	α4	-0.183
G164	α4	-0.944
A165	α4	-2.367
K166	α4	1.793
R169	α4	-6.288
E170[a]	α4	-2.862
E173	α4	-6.340
K174	α4	1.342
E51[a]	α5	-5.437
I54	α5	-3.196
T55	α5	-3.761
S56	α5	-0.739
G171	α5	-0.020
S172	α5	-0.026
M206	α5	-0.250
E207[a]	α5	-2.298
E208	α5	-2.279

[a] Negatively charged residues involved in ionic interaction with RPs.

Table S12. Inter-residues distances (Cα) of the negatively charged residues present in the putative binding site for an additional ligand molecule at the α5-α6 groove of the docked structure of human 20S in complex with **2** (Cplx_9).

**Site**	**Distances (Å)**			
	**d1**	**d2**	**d3**	**d4**
α face groove α5-α6 (I)	19.93 E29(α5)-E175(α5)	23.87 E175(α5)-D204(α6)	23.75 E204(α6)-E18(α5)	20.38 E18(α5)-E29(α5)
α face groove α5-α6 (II)	22.52 E25(α5)-E175(α5)	23.87 E175(α5)-D204(α6)	23.75 E204(α6)-E18(α5)	14.06 E18(α5)-E25(α5)
α face groove α5-α6 (III)	19.93 E29(α5)-E175(α5)	23.87 E175(α5)-D204(α6)	22.45 E204(α6)-E27(α6)	22.61 E27(α6)-E29(α5)
α face groove α5-α6 (IV)	22.52 E25(α5)-E175(α5)	23.87 E175(α5)-D204(α6)	22.45 E204(α6)-E27(α6)	16.35 E27(α6)-E25(α5)

Table S13. Kinetic parameters for the reaction of 2 and 1 with the human 20S proteasome.

2[a]		**1**[b]	
$^{1}k_{c}$ ($M^{-1}s^{-1}$)	1.3×10^{2}	$^{1}k_{on}$ ($M^{-1}s^{-1}$)	5.6×10^{4}
$^{1}k_{o}$ ($M^{-1}s^{-1}$)	2.8×10^{6}	$^{2}k_{on}$ ($M^{-1}s^{-1}$)	1.3×10^{4}
$^{1}K_{c}$ (M^{-1})	7×10^{3}	^{3}k (s^{-1})	3.2×10^{-3}
$^{1}K_{o}$ (M^{-1})	1.8×10^{7}		
$^{1}k_{off}$ (s^{-1})	0.22		
^{1}L	4×10^{-6}		
$^{2}k_{c}$ ($M^{-1}s^{-1}$)	6.8×10^{5}		
$^{2}k_{o}$ ($M^{-1}s^{-1}$)	6.75		
$^{2}K_{c}$ (M^{-1})	3.1×10^{6}		
$^{2}K_{o}$ (M^{-1})	2.5×10^{3}		
$^{2}k_{off}$ (s^{-1})	2.7×10^{-3}		
^{2}L	7.3×10^{4}		

See Eqs. S4-S7; [a]from ref 27

REFERENCES

[1] Adler, A.D.; Longo, F.R.; Shergalis, W. *J. Am. Chem. Soc.* **1964**, *86*, 3145-3149.
[2] Monod, J.; Wyman, J.; Changeux, J.P. *J. Mol. Biol.***1965**,*12*,88–118.
[3] Elsasser, S.; Schmidt, M.;Finley, D. *Methods Enzymol.***2005**,*398*,353-63.
[4] Pei, J.; Kim, B.H.; Grishin, N.V. *Nucleic Acids Res.* **2008**, 36, 2295–2300.
[5]Dauber-Osguthorpe, P.; Roberts, V.A.; Osguthorpe, D.J.; Wolff, J.; Genest, M.; Hagler, A.T. *Proteins* **1988**,*4*, 31-47.
[6] Davis, I.W.; Leaver-Fay, A.; Chen, V. B.; Block, J. N.; Kapral, G. J.; Wang, X.; Murray, L. W.; Arendall, W.B.; Snoeyink, J.; Richardson, J.S.; Richardson, D.C. *Nucleic Acids Res.* **2007**, *35*, W375-W383.
[7] ACD/Percepta, version 14.0.0, Advanced Chemistry Development, Inc., Toronto, ON, Canada, 2013, http://www.acdlabs.com.
[8] Dewar, M.J.S.; Thiel, W. *J. Am. Chem. Soc.* **1977**, *99*, 4899-4907.
[9]Fletcher, R. Unconstrained Optimization. In Practical Methods of Optimization; John Wiley & Sons: New York 1980; Vol. 1.
[10] Stewart, J.J. *J. Mol. Model.* **2013**,*19*, 1-32.
[11]MOPAC2012; Stewart Computational Chemistry: Colorado Springs, CO, USA; http://OpenMOPAC.net (2012)
[12] Baker, J. *J. Comput. Chem.* **1986**, 7, 385−395
[13] Senderowitz, H.; Guarnieri, F.; Still, W. C. *J. Am. Chem. Soc.* **1995**, *117*, 8211−8219.
[14] Ding, H.Q.; Karasawa, N.; Goddard, W.A.. *J. Chem. Phys.* **1992**, *97*, 4309–4315.
